# Designing Polyelectrolyte Microneedles Based on Borylated Poly(β-aminoester) Polymers To Enhance Transdermal pH-Controlled Delivery of Nucleic Acids

**DOI:** 10.1021/acsapm.4c00969

**Published:** 2024-07-24

**Authors:** Patricia González-Sáenz, Raúl Cosialls, Robert Texidó, Aurora Dols-Pérez, Ana Belén Cuenca, Salvador Borrós, Cristina Fornaguera

**Affiliations:** † Grup d’Enginyeria de Materials (GEMAT, Insititut Químic de Sarrià (IQS), Universitat Ramon Llull (URL), Via Augusta 390, 08017 Barcelona, Spain; ‡ BISI-Bonds/CRISOL Group, Department of Organic and Pharmaceutical Chemistry, Insititut Químic de Sarrià (IQS), Universitat Ramon Llull (URL), Via Augusta 390, 08017 Barcelona, Spain; § Institut de Bioenginyeria de Cataluña (IBEC), The Barcelona Institute of Science and Technology (BIST), C/Baldiri I Reixac 11-15, 08028 Barcelona, Spain

**Keywords:** polyelectrolytes, multilayered coating, microneedles, borylated poly(β-aminoester), gene delivery

## Abstract

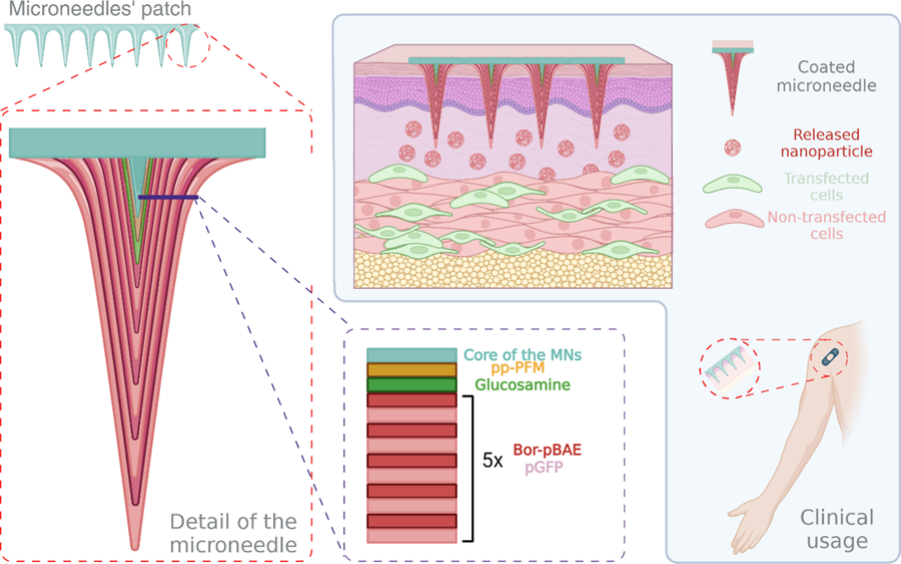

The use of transdermal delivery for nucleic acid administration is an interesting approach to overcoming limitations of systemic administration routes, such as first-pass effects, the painful needle injection, or their poor biodistribution. Thus, the use of a microneedle-based patch could represent a turning point for nucleic acid delivery, thanks to the possibility of self-administration of the actives in a painless and easy procedure. However, the design of transdermal systems with a higher degree of precision release is a clear need that has not been fully resolved. Committed to tackling this challenge, we present here a microneedle patch that involves a smart delivery system supported by the well-established ability of boronic acid to interact with carbohydrates in a pH-dependent manner. This system builds up a multilayer structure over a solid microneedle platform whose surface has been modified to immobilize glucosamine units that are able to interact with an oligopeptide-end terminated poly(β-aminoester) that presents a 4-carboxy-3-fluorophenylboronic acid (Bor-pBAE). Thus, sequential layers of the Bor-pBAE and plasmid DNA have been assembled, thanks to the ability of the polymer to interact with the nucleic acid at a basic pH and then gradually release the plasmid under two different conditions of pH (the physiological pH = 7.4 and the acidic pH = 5.1). We set up the design and implementation of this first proof of concept while demonstrating microneedles' safety and functionality. Additionally, we have shown the efficacy of the construct to express the encoded genes in model cell lines. In conclusion, we have established the basis to confirm that this generation of borylated poly(β-aminoesters) holds great promise as a transdermal local nucleic acid delivery system.

## Introduction

Surface functionalization by multilayered thin films has been used for designing delivery systems that are able to modulate the dose deposited as well as the release of biomolecules in a controlled manner.^1,2^ The idea is based on depositing thin films following the layer-by-layer (LbL) approach which determines the thickness of the film together with the physicochemical properties and subsequent application of interest.^3−5^ Therefore, taking advantage of the properties of the biomolecules immobilized over the surfaces, the LbL technique has been applied in the biomedical field for the creation of drug/gene delivery systems, biosensing, or regenerative medicine, among others.^5,6^

From all those applications, genetic-based therapies hold great promise for prophylactic vaccination, cancer immunotherapies, and tissue engineering. Nevertheless, the number of gene-based products on the market is still limited, due to the weaknesses that delivery systems and administration routes still have.

Concerning delivery systems, we developed some years ago a biocompatible and biodegradable synthetic polymer family, oligopeptide-end-modified poly(β-aminoesters) (OM-pBAEs), with enhanced capacity to complex a huge range of nucleic acids of different lengths, including viral vectors, into small nanometric polyplexes, and promote the expression of the encapsulated nucleic acid when systemically administered in preclinical models.^7−12^ However, systemic administration can run with off-target effects, usually requires higher doses of administration, and, more importantly, the stability of the nanoparticles in biological fluids can be compromised due to biocorona formation.^13,14^ Thus, local administration could be an interesting alternative. Skin is the ideal organ for such a delivery beyond skin diseases (i.e., skin cancers, infections, and wounds), such as immune-based diseases (i.e., immunotherapies for cancer, infectious diseases, or autoimmune diseases), thanks to the presence of numerous immune cells, such as Langerhans cells, one of the most efficient antigen-presenting cells, high vascularization, and lymph ducts.^15−17^ The downside is that, at the same time, the skin is the main barrier of our body preventing the penetration of exogenous compounds. In this context, microneedle patches or arrays (MNs) capable of piercing and penetrating the stratum corneum to achieve a minimally invasive, painless, patient-friendly transdermal delivery have been proposed as an appealing approach.^18,19^ Using micron-sized needles, first-pass effects of the intradermal route can be avoided, and thus, delivery of active principles directly to skin-permeable regions is achieved. Additionally, the possible self-administration is an advantage for low-income countries.^16,20−23^ Thus, there are huge expectations for their promising application for many diseases, and, among them, vaccination and more specifically tumor immunotherapy applications.^24^

Biomaterials have been used as solid substrates to design MNs, incorporating the genetic material directly in their formation or by performing different coating formulations. While in the first case, the genetic material is degraded in contact with the substrate, in the second one, problems of burst release still hamper their use. To address this limitation, inspired by the LbL approach, we have developed an innovative microneedle patch-based smart delivery system. Our approach leverages the well-established ability of boronic acid to interact with carbohydrates,^25,26^ allowing for a stable yet pH-reversible coating of the polymeric vector/nucleic acid complex onto the surface of the microneedles. This approach provides a higher degree of control over the release of the nucleic acid at two different pHs, thereby enhancing the efficacy of the delivery system. The system builds upon a solid microneedle whose surface has been modified with plasma-polymerized pentafluorophenyl methacrylate (pp-PFM) to immobilize a monolayer of glucosamine. Next, the sugar interacts with a new OM-pBAE that presents a series of units of 4-carboxy-3-fluorophenylboronic acid (**Bor-pBAE**). Thanks to the interaction between glucosamine and boronic acid, the newly developed **Bor-pBAE** can be immobilized to form a stable layer on the surface of the microneedles. Taking advantage of this property, we constructed a multilayer structure based on polyelectrolyte assembly consisting of alternating layers of **Bor-pBAE** and nucleic acids. This approach provides a highly controlled and efficient method to deliver the nucleic acid ensuring that the release takes place gradually, thanks to the sustained generation of the free boronic acid form of the **Bor-pBAE** polymer at both pHs.

pBAEs had been previously used for MN formulation, as constituents of nucleic acid-loaded nanoparticles encapsulating nucleic acids.^15,17,20,22,27^ However, as far as we know, this is the first time that a poly(β-aminoester) chemically modified with boronic acid moieties has been used for MN formulations. For that, we have used our OM-pBAE modified with borylated fragments (organic trifluoroborates) recently developed by our group.^28^ Moreover, it is important to note that, in all previous studies involving the use of pBAEs as delivery systems in microneedles, the design of the microneedles was somehow sophisticated, thus hampering the transference to the market. Through the results of the present study, we demonstrated that our multilayered MN platform could represent a promising alternative to the delivery of nucleic acids.

## Experimental Section

### Materials

Metallic microneedles were purchased from KoyBeauty Dermapen (cartridges of 12 microneedles). All commercially acquired reagents were used as received unless indicated otherwise. Pentafluorophenyl methacrylate (PFM) was purchased from Apollo Scientific Ltd. (Stockport, U.K.). d-(+)-Glucosamine hydrochloride was obtained from Sigma-Aldrich (Missouri, USA). Ethanol and acetone were supplied by Scharlab (Barcelona, Spain). Oxygen 5.0 and argon 5.0 were obtained from Carburos Metalicos (Cornellà de Llobregat, Barcelona, Spain). 4-Carboxy-3-fluorophenylboronic acid was purchased from FluoroChem (United Kingdom). 2-Fluoro-4-(4,4,5,5-tetramethyl-1,3,2-dioxaborolan-2-yl)benzoic acid (**FPBpin-COOH**)^29^ was prepared from 4-carboxy-3-fluorophenylboronic acid according to a literature procedure with slight modifications. Cyanine 5-NHS ester and Cyanine 3-NHS ester were supplied by Lumiprobe (Hanover, Germany).

Chemical reactions requiring an inert atmosphere were conducted under an argon atmosphere using standard Schlenk line techniques. Thin layer chromatography (TLC) was performed using Merck plastic-backed plates of TLC Silica gel 60 F254; the plates were revealed using UV light at 254 nm or by staining using potassium permanganate. Standard flash column chromatography was accomplished using Merck silica gel (60 Å pore size, 70–230 μm mesh size).

Spectroscopic experiments for the characterization of compounds were carried out at the Structural Determination facility of IQS on a Varian 400-MR spectrometer (400 MHz for ^1^H, 100.5 MHz for ^13^C, 376 MHz for ^19^F, and 128 MHz for ^11^B). Chemical shifts (δ_Η_) are quoted in parts per million (ppm) and referenced to the appropriate NMR resonance, which for ^1^H measurements would correspond to the residual portion component of the deuterated solvent. The ^19^F and ^11^B chemical shifts are referenced relative to CFCl_3_ and BF_**3**_·Et_**2**_O resonance at 0.00 ppm, respectively. 2D-NMR experiments COSY, HSQC, and HMBC were used where necessary in assigning NMR spectra. Spin–spin coupling constants (*J*) are reported in Hertz (Hz). Results of the detailed characterization are found in the Supporting Information.

pGFP (DNA plasmid encoding for green fluorescent protein) was produced using *E. coli* (DH5α competent cells) cultures that were transformed by the plasmid and amplified in LB (Luria–Bertani) medium containing specific antibiotic overnight in a shaker at 37 °C. Then, bacteria were harvested and plasmids were extracted in a big scale (Gigaprep) and purified using a plasmid kit of ion exchange columns called the NucleoBond Xtra Giga kit for transfection-grade plasmid DNA (Macherey-Nagel, Düren, Germany) according to the procedure provided by the manufacturer. DNA concentration was determined using a spectrophotometer NanoDrop applying an absorbance of 260 nm and an extinction coefficient of 50 (ng/μL)^−1^ cm^–1^.

### Cell Culture

The B16F10 cell line has an epithelial phenotype with a spindle shape, and these cells are isolated from the skin tissue of a mouse with melanoma. Those cells are adherent, and they grow in DMEM (L0101, Dulbecco’s modified Eagle’s medium, Biowest, France) supplemented with 10% fetal bovine serum (FBS) (Avantor, Pennsylvania, USA), 1% l-glutamine (X0550, Biowest, France), and 1% penicillin/streptomycin (25030081, Gibco, Thermo Fisher Scientific, Waltham, MA, USA). The conditions of growth were at 37 °C and 5% CO_2_. When they reached around 70–80% confluence, a 1:5 or 1:10 passage was performed using trypsin/EDTA (L0931, Biowest, France).

### Methods

#### Nanoparticles’ Formulations

Two different formulations of nanoparticles were prepared following the molar ratio 25:1 (polymer:nucleic acid) in a mix of pGFP (concentration of 0.5 mg·mL^–1^) and the already synthesized Bor-pBAE (12.5 mg·mL ^–1^). The molar ratio was 50:1 (polymer:nucleic acid) in a mix of pGFP (concentration 0.5 mg· mL^–1^) with the polymer Bor-pBAE (25 mg·mL ^–1^). Both Bor-pBAE and pGFP were dissolved in a solution of sodium acetate 12.5 mM at pH = 8. The protocol was performed by pipetting up and down the nucleic acid over the Bor-pBAE solution followed by incubation for 30 min at room temperature. Afterward, those polyplexes were nanoprecipitated in the same volume of sterilized water.

#### Dynamic Light Scattering (DLS)

Physicochemical properties of nanoparticles’ formulations were determined by DLS (Malvern Instruments Ltd., United Kingdom, 4 mW laser) with a detection angle of 173° and a laser of 633 nm. For the measurements, nanoparticles’ formulations had a concentration of 0.25 mg mL^–1^ of pGFP for determining the hydrodynamic size while they had a final concentration of 0.025 mg mL^–1^ for measuring the surface charge. All those measurements had been performed three times with 20 runs per measurement.

#### Determination of Nucleic Acid Encapsulation by Electrophoretic Mobility Shift Assays

The ability of nanoparticles to encapsulate pGFP at different polymer ratios was studied with the electrophoretic mobility of polymers, which was measured on agarose gels (1% of agarose w/v) in Tris–acetate–EDTA (TAE) 1× buffer containing GelRed Nucleic Acid Gel Stain (Biotium, Fremont, California, USA). The electrophoresis mixture was added to the tray, and the gel was allowed to solidify for 30 min. Then the electrophoresis tray was placed into the TAE 1× electrophoresis cuvette. Then, samples were loaded and were run for 1 h at 80 V (Apelex PS 305, France). Finally, encapsulated and DNA-free bands were visualized by UV irradiation.

#### Encapsulation Efficiency Test of Nanoparticles

To quantify the free (nonencapsulated) double-stranded DNA, the PicoGreen quantification assay kit was used following the manufacturer’s instructions.

#### Plasma Polymerization of PFM

First, to perform plasma polymerization over substrates, stainless-steel plates and microneedles were washed with Milli-Q water and then with ethanol, dried under nitrogen, and stored in an argon atmosphere. Plasma polymerization of the monomer PFM was performed using a plasma stainless-steel reactor, manufactured by the GEMAT group (Barcelona, Spain).^30^ This reactor has an external chamber (41.6 cm × 25.5 cm) working as a ground electrode and an aluminum plate as a radio frequency (RF) electrode used to hold the samples for polymerization. This RF electrode is connected to an RF pulse generator (13.56 MHz) via a matching box. The entry of gases and monomers is regulated by a standard manifold, and over the chamber, there is a tree of needle valves working as a gas collector. A vacuum gauge controller performs the monitoring of the pressure (MKS PDR900, Andover, MA, USA) which is connected to a cold cathode/MicroPirani vacuum transducer (MKS 972 DualMag) at the center of the reactor. The system possesses a nitrogen cold trap and a chemical trap with active carbon to prevent nonreacted monomers from reaching the pump (Trivac D 16BCS/PFPE Leybold, Cologne, Germany). The procedure of plasma polymerizations for those surface modifications was carried out as described in previous papers by our group.^31,32^

#### Immobilization of Reducing Saccharides

Once the pp-PFM film was grafted over the surfaces, the samples were incubated in the presence of glucosamine 1 M at pH = 8 to perform the immobilization of this reducing sugar on pp-PFM for functionalizing surfaces (Figure S31). Then, samples were stored under an argon atmosphere.

#### Synthesis of Borylated Poly(β-aminoesters)

##### Synthesis of **C6**

A 10 mL round-bottom flask was charged with a mixture of monomers: 5-amino-1-pentanol (1.0 g, 10 mmol, 0.5 equiv), 6-hexylamine (1.3 mL, 10 mmol, 0.5 equiv), and 1,4-butanediol diacrylate (4.5 mL, 24 mmol, 1.2 equiv) and was allowed to stir at 90 °C for 20 h. Subsequently, the triblock copolymer was isolated as a yellowish viscous oil, 6.2 g, 91% yield. According to ^1^H NMR, the new polymer exhibited, on average, a total of three repeating amino-1-pentanol units and three additional units of hexylamine chains (Figure S2). Once synthesized, the **C6** pBAE was transferred to a screw-cap vial and stored at 4 °C.

##### Synthesis of **C6-FPBpin**

In a round-bottom flask, and under an argon atmosphere, poly(β-aminoester) **C6** (600 mg, 0.30 mmol, 1 equiv) and the fluorinated aryl boronate derivative **FPBpin-COOH** (239 mg, 0.90 mmol, 3 equiv) were dissolved in anhydrous CH_2_Cl_2_ (8 mL) in the presence of DCC (185 mg, 0.90 mmol, 3 equiv) and DMAP (11 mg, 0.09 mmol, 0.3 equiv). The mixture was then stirred at room temperature for 18 h. The obtained suspension was filtered, and the filtrate was evaporated to dryness, redissolved in Et_2_O (100 mg/mL), and stored overnight at 4 °C for the precipitation of residual traces of dicyclohexylurea. The mixture was filtered once more over a 0.45 μm nylon filter, and the filtrate was concentrated to dryness. According to ^1^H NMR, the obtained polymer (**C6-FPBpin**), a yellow dense oil (686 mg, 82% isolated yield), exhibited an 84% of the initial 5-amino-alcohol chains esterified with the corresponding aryl pinacol boronate. ^1^H NMR (CDCl_3_, 400 MHz) δ 7.86 (t, *J* = 7.3 Hz, 3H), 7.56 (d, *J* = 7.7 Hz, 3H), 7.49 (d, *J* = 11.1 Hz, 3H), 6.38 (dd, *J* = 17.3, 1.5 Hz, 2H), 6.10 (dd, *J* = 17.3, 10.4 Hz, 2H), 5.81 (dd, *J* = 10.4, 1.5 Hz, 2H), 4.30 (t, *J* = 6.6 Hz, 5H), 4.20–4.14 (m, 4H), 4.14–4.00 (m, 25H), 2.74 (t, *J* = 7.2 Hz, 24H), 2.46–2.33 (m, 36H), 1.85–1.57 (m, 38H), 1.57–1.35 (m, 20H), 1.32 (s, 37H), 1.29–1.22 (m, 18H), 0.90–0.82 (m, 10H). ^13^C NMR (CDCl_3_, 100 MHz) δ 172.7, 166.2, 164.7, 162.7, 160.1, 131.3, 130.8, 129.9, 129.8, 128.5, 122.7, 122.5, 84.4, 65.4, 64.1, 63.9, 53.8, 53.6, 49.3, 32.6, 31.9, 28.6, 27.2, 27.1, 27.0, 25.4, 25.0, 23.8, 22.8, 14.2. ^19^F NMR (CDCl_3_, 376 MHz) δ −111.4. ^11^B NMR (CDCl_3_, 128 MHz) δ 30.2. FTIR (ATR) cm^–1^: 2931.4, 2857.1, 1728.9 (C=O st), 1406.9, 1357.7, 1268.0, 1169.7, 1141.7, 1096.4, 1034.7, 965.2. Spectra are shown in Figures S4–S7.

##### Deprotection of Boronate **C6-FPBpin**: Access to Free Boronic Acid-Modified Poly(β-aminoester) **C6-FPBA**

To an oven-dried 25 mL Schlenk tube containing a solution of poly(β-aminoester) **C6-FPBpin** (200 mg, 0.08 mmol, 1 equiv) in anhydrous CH_2_Cl_2_ (4 mL), methyl boronic acid (68 mg, 1.14 mmol, 15 equiv) and trifluoroacetic acid (TFA, 0.1 mL) were added under an argon atmosphere. The resulting suspension was allowed to stir at room temperature for 24 h. At this point, the reaction mixture was evaporated to dryness using the Schlenk line vacuum followed by a further drying step of 2 h at room temperature. The obtained residue was dissolved in DMSO (2 mL) and treated with a mixture of acetone:diethyl ether 1:4 (20 mL). This treatment induced the precipitation of the desired polymer. The mixture was centrifugated, the supernatant was removed, and the pellet was washed with acetone:diethyl ether 1:4 (10 mL × 3). The pure polymeric material was next dried under vacuum overnight at 60 °C. According to ^1^H NMR, the obtained poly(β-aminoester) (**C6-FPBA**), a yellow dense oil (227 mg, 96% isolated yield), exhibited an 87% of the 5-amino-alcohol chains esterified with the 2-fluoro phenyl boronic acid derivative. ^1^H NMR (DMSO-*d*_*6*_, 400 MHz) δ 7.83 (t, *J* = 7.4 Hz, 4H), 7.68 (d, *J* = 7.7 Hz, 4H), 7.61 (d, *J* = 11.7 Hz, 5H), 6.32 (d, *J* = 17.3 Hz, 2H), 6.16 (dd, *J* = 16.4, 11.1 Hz, 2H), 5.94 (d, *J* = 10.3 Hz, 2H), 4.29 (t, *J* = 6.5 Hz, 10H), 4.20–3.94 (m, 48H), 3.45–3.23 (m, 44H), 3.21–2.99 (m, 22H), 2.97–2.74 (m, 43H), 1.85–1.55 (m, 80H), 1.49–1.36 (m, 11H), 1.36–1.19 (m, 34H), 0.94–0.77 (m, 16H). ^13^C NMR (DMSO-*d*_*6*_, 100 MHz) δ 170.1, 165.5, 163.8, 161.8, 159.2, 158.8, 158.5, 131.5, 130.6, 129.8, 128.3, 121.8, 121.6, 119.4, 64.7, 64.1, 63.7, 60.3, 52.6, 47.8, 30.7, 28.2, 27.6, 25.6, 24.7, 24.6, 23.0, 22.5, 21.9, 13.8. ^19^F NMR (DMSO-*d*_*6*_, 376 MHz) δ −112.5. ^11^B NMR (DMSO-*d*_*6*_, 128 MHz) δ 31.14. FTIR (ATR) cm^–1^: 2958.4, 2872.6, 1727.0 (C=O st pBAE backbone), 1669.2 (C=O st trifluoroacetate), 1406.9,1269.0, 1176.4, 1129.2, 969.1. Spectra are shown in Figures S16–S19.

##### Synthesis of Oligopeptide-Modified Poly(β-aminoester) **C6-FPBA**-C_2_R_6_ (Bor-pBAE)

A 1.5 mL Eppendorf tube was charged with 200 μL from a solution of **C6-FPBA** in DMSO (100 mg/mL, 4 μmol, 1 equiv), 200 μL from a solution of the hydrochloric form of the tetrapeptide Cys-(Arg)_3_ (CR_3_·HCl) dissolved in DMSO (100 mg/mL, 27 μmol, 7 equiv), and 10 μL of Et_3_N (72 μmol, 18 equiv). The mixture was homogenized using vortex and allowed to stir at 25 °C for 24 h. When the Michael addition reaction was deemed complete by ^1^H NMR, the reaction mixture was treated with a mixture of acetone:Et_2_O 3:7 (10 mL). This treatment induced the precipitation of the oligopeptide-modified polymer. The mixture was centrifugated, and the pellet was washed with acetone:Et_2_O 3:7 (2 × 5 mL). The residue was then dissolved in DMSO (100 mg/mL) and purified by the previous precipitation protocol 2 more times. The pure polymeric material was next dried under vacuum overnight. The final OM-pBAE was isolated as a white solid, 34 mg, 85% yield. ^1^H NMR (DMSO-*d*_*6*_, 400 MHz) δ 8.99 (d, *J* = 7.4 Hz, 10H), 8.52–8.16 (m, 35H), 8.03 (br s, 11H), 7.97–7.78 (m, 31H), 7.68 (d, *J* = 7.7 Hz, 5H), 7.62 (d, *J* = 11.8 Hz, 6H), 4.43–4.17 (m, 43H), 4.17–3.90 (m, 63H), 3.54–3.21 (m, 115H), 3.20–2.89 (m, 137H), 2.88–2.53 (m, 68H), 1.89–1.33 (m, 245H), 1.24 (br s, 36H), 0.84 (m, 16H). ^13^C NMR (DMSO-*d*_*6*_, 100 MHz) δ 173.6, 171.6, 171.4, 170.9, 170.7, 167.4, 167.2, 163.8, 161.8, 159.2, 157.1, 130.6, 129.9, 121.7, 119.41, 64.9, 63.8, 60.5, 52.7, 52.6, 52.4, 52.1, 51.3, 48.1, 34.1, 31.0, 30.8, 29.0, 28.2, 27.8, 26.2, 26.0, 25.3, 25.0, 24.8, 22.9, 22.05, 15.2, 13.9. ^19^F NMR (DMSO-*d*_*6*_, 376 MHz) δ −112.5. ^11^B NMR (DMSO-*d*_*6*_, 128 MHz) δ 27.05 (B(OR)_2_), −3.26 (B(OR)_3_). FTIR (ATR) cm^–1^: 3162.8, 2955.5, 1728.9 (C=O st pBAE backbone), 1647.9 (C=O st trifluoroacetate), 1548.6, 1403.0, 1186.1, 993.2. Spectra are shown in Figures S4–S7.

#### Synthesis of Bor-pBAE Polymer Labeled with Cy5

Cyanine 5-NHS ester (Cy5) labeling of Bor-pBAE was performed by mixing the Bor-pBAE in DMSO with triethylamine and Cy5. This solution was stirred for 20 h and incubated in a water bath under a controlled temperature of (25 ± 2) °C. Then, the product was precipitated with a mix of diethyl ether:acetone (7:3, v/v) and centrifuged twice at 4000 rpm for 10 min to get rid of the solvent. Finally, the product was dried under vacuum and dissolved in DMSO at a final concentration of 100 mg/mL.

#### Polyelectrolyte Multilayer System Assembly Analysis and Study of the Multilayered Release

Once the first two films (pp-PFM and glucosamine) had been deposited over the surface of microneedles, a polyelectrolyte multilayer (PEM) system was assembled. This PEM system is composed of alternate polycation/polyanion films, respectively, of Bor-pBAE and pGFP. Following the principle of depositing films LbL, films of Cy5-Bor-pBAE (2 mg/mL) and pGFP (1 mg/mL) were alternatively deposited over glucosamine. Both were prepared in sodium acetate (Sigma-Aldrich, St. Louis, Missouri, USA) 100 mM, pH = 8, and surfaces were dipped in these solutions for 5 min.

Afterward, a second incubation at 37 °C was performed mimicking the procedure described by Zhang et al.^33^

Additionally, the analysis of the PEM assembly and release was studied using quartz crystal microbalance with dissipation (QCM-D).

QCM-D technology (Q-Sense E1, Sweden) was used to characterize the interactions of the PEM system composed of **Bor-pBAE** and pGFP biomolecules. This technique is suitable for monitoring the frequency (*f*) and dissipation (*D*) of an oscillating sensor for studies of changes in mass or mechanical properties. It is suitable for analyzing the adsorption/desorption properties, the viscoelasticity of thin films, and swelling properties. The sensor is a piezoelectric quartz crystal sensor (Q-Sense) with polished gold electrodes that have a root-mean-square roughness of less than 3 nm, a diameter of 13 mm, and an effective area of 5 mm diameter.

Changes in the oscillation frequencies of a piezoelectric crystal are registered when mass is deposited over. These parameters are related following the Sauerbrey equation (eq 1).
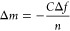
1

In eq 1, the mass sensitivity constant is known as *C* (17.7 ng·cm^–1^ ·Hz^–1^ for a 5 MHz sensor) and *n* (1,3,5,...) is the overtone number.

Moreover, the dissipation signal coming from the oscillating sensor is related to the ratio between the dissipated energy and the stored energy (eq 2). This parameter studies the viscoelastic properties of the film deposited over the sensor.
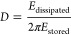
2

#### Flow Cytometry Analysis of Nanoparticles

The analysis of the released media coming from the multilayered coated metallic microneedles was performed using the Cytoflex S cytometer (Beckman Coulter, California, USA). In this experiment, multilayered coated microneedles (when performing the PEM assembly protocol, the Bor-pBAE polymer used was labeled with Cy5 fluorophore) were incubated at 37 °C for 1 h under two different pH conditions (pH = 7.4 and pH= 5.1). The released medium was analyzed by flow cytometry. The gated region of Cy5-positive released nanoparticles was determined considering a positive control of nanoprecipitated Cy5-Bor-pBAE nanoparticles, and the size of those particles was established comparing with size regions displayed by known size from commercial reference nanofluorobeads (MegaMix Plus FSC 50 test, B91907; MegaMix Plus SSC 50 test, B91906, Beckman Coulter, California, USA).

As representative controls, Bor-pBAE nanoprecipitated particles and Cy5-positive labeled Bor-pBAE nanoprecipitated particles were performed.

#### In Vitro Transfection Efficiency and Cytotoxicity

Transfection experiments were performed in 96-well plates at an initial seeding density of 20,000 cells/cm^2^ supplemented with DMEM. Cells were grown for 24 h, after which transfection assays were performed. To check the presence of positive cells transfected, fluorescence images were taken after 48 h using a microscope (Zeiss Axioscope 5, Jena, Germany). Additionally, this was quantified by flow cytometry (NovoCyte 451161226547, Agilent Technologies, Santa Clara, California, USA). In flow cytometry experiments, cell media were aspirated, and cells were washed with warm PBS 1×. Then, cells were trypsinized (ratio 1:4) and fixed with 4% paraformaldehyde in DMEM.

Regarding cytotoxicity studies, MTT (3-(4,5-dimethylthiazol-2-yl)-2,5-diphenyltetrazolium bromide)^34^ analysis has been performed to determine cell viability. After 24 h of incubation in the presence of FBS, 0.5 mg/mL of MTT were added at each well and incubated at 37 °C for 2 h. After this time, media was removed and DMSO was added to dissolve the formazan crystals. Absorbance was quantified at 565 nm, using a plate reader (SpectraMax M5 luminometer, Molecular Devices). Results were expressed as relative viability percentage compared to negative control cells (100% viability).

#### Procedure of Labeling pGFP with Cyanine 3-NHS Ester (Cy3)

Cyanine 3-NHS-ester (Cy3) labeling with pGFP was performed following the manufacturer’s instructions of Label IT Tracker TM Intracellular Nucleic Acid Localization kit. Regarding the purification process, the product was centrifuged at 14,000*g*, 4 °C for 15 min. After that, the pellet was cleaned with ethanol (70%) and centrifuged again. Then, the supernatant was discarded, and the product was dried to finally be resuspended in sterilized water. To quantify the product, a NanoDrop spectrophotometer was used (DeNovix DS-11 series, DeNovix Inc., USA).

#### Confocal Scanning Fluorescent Microscopy

Qualitative studies of cell uptake and transfection were carried out by a Leica TCS SP8 laser-scanning confocal spectral microscope (Leica Microsystems Heidelberg, Manheim, Germany) with argon and HeNe lasers attached to a Leica DMi8 S platform inverted microscope. For visualization of the nanoparticles' uptake and transfection, images were acquired using an APO 40× objective lens. Numerical aperture 1.4; 405, 488, 528, and 633 nm laser lines, acoustic beam splitter as a beam splitter, emission detected in the ranges of 410–430, 500–520, 535–580, and 645–660 nm, and the confocal pinhole set at 1 Airy unit; nuclei were stained by 5 min incubation with DAPI and further mounting the copper slices in a glass slide.

#### Skin Insertion Studies

The insertion studies were tested using ex vivo skin explants from cadaveric porcine skin. Microneedles were coated following the coating and PEM assembly protocols previously described, and then they were inserted in the skin using a dynamometer applying a force of 60 N for 10 min. After this method was performed, the pierced skin samples were analyzed using a fluorescence microscope (Zeiss Axioscope 5, Cambridge, UK) to check the deposition of the treatment within the holes of the needles performed. Additionally, skin-pierced tissues were embedded in paraffin blocks to be subsequently analyzed histologically. The paraffin blocks were perpendicularly sectioned by a microtome (to analyze the depth of insertion of the microneedles) into thin slices to be placed into slides and being stained by hematoxylin and eosin.

#### Statistical Analysis

Data are represented by the mean ± standard deviation from at least three independent determinations. Significant differences were analyzed by the Kruskal–Wallis test followed by Dunn’s multiple comparison test for more than two data comparisons, while the Mann–Whitney *U* test was applied for one-to-one sample comparison, using the GraphPad Prism software version 8.0 (GraphPad Software, San Diego, CA, USA), and a *p*-value below than 0.05 (*p < 0.05, **p < 0.01, ***p < 0.001) was considered statistically significant.

## Results and Discussion

### Design, Synthesis, and Functionalization of the New **C6-FPBA-C_2_R_6_** (**Bor-pBAE**)

**Scheme 1:**
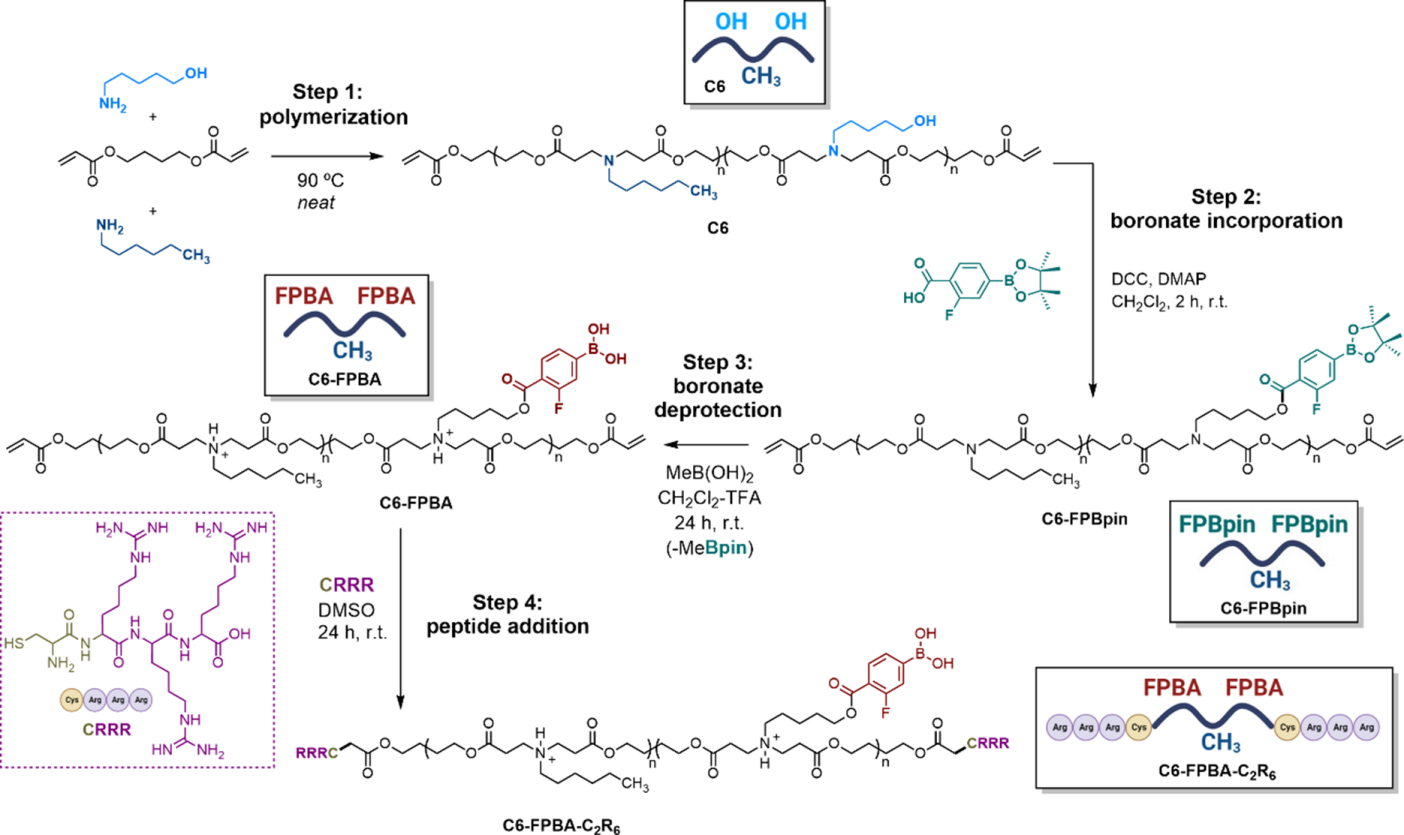
Synthetic Route toward the Preparation of **C6-FPBA-C2R6** (**Bor-pBAE**)

As mentioned, we sought the preparation of a new 4-carboxy-3-fluorophenylboronic acid-modified poly(β-aminoester) based on a **C6**-type pBAE (Scheme 1, step 1). We chose this fluorinated boronic acid fragment under the hypothesis that this borylated moiety could have the appropriate electronic deficiency to induce a clear interaction with the glucosamine sugar at pH = 8, the pH at which the coating takes place. It is important to consider that the stability of the boronic acids/sugar adducts is usually directly related to the p*K*_a_ of the boronic acid itself and the pH of the medium in which the interaction is occurring. A couple of recent studies^35−37^ have illustrated the potential of this fluorinated phenyl boronic acid fragment in glucose-responsive insulin transdermal delivery systems. In the context of gene delivery, these types of borylated units have been nicely studied by Professor Kataoka’s group;^26,38,39^ however, to the best of our knowledge, they have never been applied to a microneedle-based transdermal system. Interestingly, in most of the cases, the boronic acid fragment employed by their team is linked to the corresponding polymeric vector through the formation of an amide. On the basis of the **C6** poly(β-aminoester) structure, however, the most logical strategy would be the conjugation of the boronic fragment to the amino alcohol lateral chains of the polymer using the formation of an ester. Before undertaking the synthesis of the new borylated pBAE, we considered that the p*K*_a_ determination of a model fluorinated boronic acid derivative esterified with *n*-butanol might be useful data (see Figures S1–S11 for details). Hence, the resulting p*K*_a_ turned to be 6.8 (Figure S12), a slightly lower p*K*_a_ (7.2)^40^ when compared to the corresponding amide derivates used in the literature, a fact that allows us to predict a good interaction with the glucosamine attached to the microneedle in the first step of the LbL at the desired pH.^26^

Once the boronic acid fragment was validated, we undertook the synthetic sequence of the new **C6-FPBA-C**_**2**_**R**_**6**_ (**Bor-pBAE**) that implies the first step of polymerization followed by boronic acid incorporation-deprotection and, in the last step of the synthesis, peptide double end-capping by Michael addition.

The **C6** poly(β-aminoester) was prepared according to a previously described protocol (Scheme 1, step 1),^10^ and the number of hydroxyl chain units was estimated based on the ^1^H NMR integration (Figures S13 and S14, from SI). As an efficient route to append the fluoroboronate units to the **C6** poly(β-aminoester), we sought the esterification of all the hydroxyl groups present at the lateral chains of the polymer with the fluoro carboxylic pinacol boronate (**FPBpin-COOH**) derivatives^29^ (Scheme 1, step 2). Careful analysis of the ^1^H NMR data after the reaction revealed a significant downfield of the C*H*_2_–OH protons (from 3.62 to 4.30 ppm) because of the esterification process. At the same time, the presence of a signal at 1.32 ppm indicated the unequivocal incorporation of the pinacol boronate unit in the polymer. Based on the NMR analysis, an 84% esterification degree of the total hydroxyl groups on the starting **C6** was accomplished, and the alcohol-modified/alkyl chains ratio was maintained at 1:1 (see Figures S15–S19, from SI). Next, we proceeded to transform the boronic ester groups in **C6-FPBpin** to their corresponding free boronic acid form. Initial attempts to perform this deprotection reaction using classic methodologies such as oxidative cleavage with sodium periodate^41^ proved unsuccessful, most probably due to the low solubility of the unprotonated form of the polymer in the basic media of the oxidative reaction. Then we turned our attention to a new acid-assisted transesterification strategy recently reported by Klein’s laboratory.^42^ To this end, a CH_2_Cl_2_ solution of **C6-FPBpin** was exposed to an excess of methylboronic acid in the presence of TFA (Scheme 1, step 3). During a period of 24 h, all the volatiles, including the pinacol methyl boronate byproduct, were removed under vacuum. The absence of the characteristic pinacol singlet (1.32 ppm) in ^1^H NMR (Figure S4) together with the presence of a signal at 31.4 ppm in ^11^B NMR (Figure S7) demonstrated both: the correct deprotection of the boronate fragment and the persistence of the boronic acid unit in the final **C6-FPBA** (see Figures S20–S24, from SI). Additionally, the shift in DMSO-*d*_*6*_ of the N–C*H*_2_CH_2_–(C=O) signal from 2.36 (superposed with the N–CH_2_C*H*_2_CH_2_ protons) to 3.34 ppm evidenced the protonated state of the amine units of the poly(β-aminoester) (see Figures S13 and S25, from SI), **C6** and **C6**·TFA pBAEs in DMSO-*d*_*6*_), and the −74.1 ppm signal in ^19^F NMR along with the 1669.2 cm^–1^ carbonyl band in infrared spectroscopy confirmed the presence of trifluoroacetate as a counterion. This strategy yielded the desired **C6-FPBA** in an excellent 96% yield and, under the presence of TFA, the B(OH)_2_ group was found in its protonated form thus avoiding the boronic–boronic interactions and increasing the solubility in DMSO. Finally, the acrylate-ended **C6-FPBA** was end-capped with the tetrapeptide Cys-(Arg)_4_ (CR_3_·HCl) employing a conjugated Michael addition in the presence of triethylamine in DMSO (Scheme 1, step 4). The analysis of the resulting polymer by ^1^H NMR revealed the disappearance of the diacrylate signals (6.4–5.8 ppm) together with the presence of the peptide bands confirming the formation of the **C6-FPBA-C**_**2**_**R**_**6**_ poly(β-aminoester), from now on **Bor-pBAE** (Figures S26–S30, from SI).

**Figure 1:**
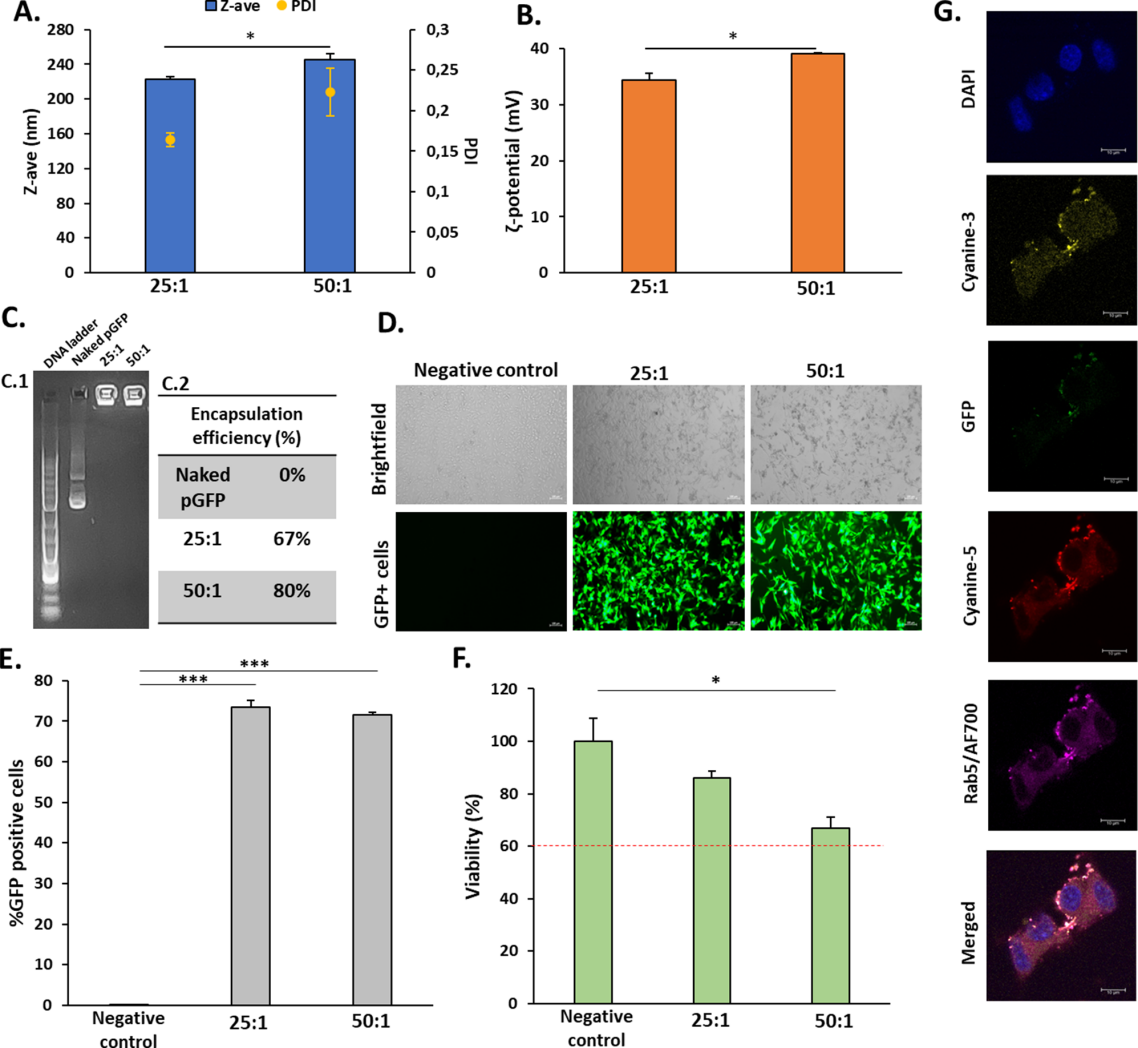
Encapsulation and transfection efficiency of Bor-pBAE nanoparticles. (A) Hydrodynamic diameters (solid bars) and PDI (dots) of polyplexes measured by DLS. (B) Surface charge in mV of polyplexes measured by DLS. (C) Encapsulation efficiency of both formulations analyzed by EMSA (C1) and by an encapsulation efficiency kit (C2). (D, E) Cellular transfection degree after 48 h based on the GFP reporter plasmid expression. Values are expressed as mean ± standard deviation. (F) Normalized viability of B16F10 cells by an MTT assay. (G) Representative confocal micrographs of the NP uptake, visualized after 8 h of incubation of NPs with cells (complete cell trafficking in SI, Figure S37). Statistical analysis (*p < 0.05; ***p < 0.001).

Taking advantage of the exceptional properties of poly-β-aminoesters to act as delivery vectors of drugs and nucleic acids^7−9,12,13^ together with the pH-responsive capacity of boronic moieties to be complexed, we wanted to test the ability of this newly synthesized polymer for performing nanoparticles to be able to act as gene delivery systems. Therefore, we established two formulations with different ratios (25:1 and 50:1) of polymer (Bor-pBAE):nucleic acid (pGFP: plasmid that codifies for the green fluorescent protein). As observed in Figure 1A, formulations exhibited a hydrodynamic size of 223 nm (25:1) and 246 nm (50:1) respectively. Regarding the Z-potential, nanoparticles displayed a positive potential of 34 (25:1) and 39 mV (50:1), thanks to their end-capped arginines (Figure 1B). Then, the test of encapsulation efficiency of pGFP was performed (Figure 1C.2) in both formulations demonstrating a 67% encapsulation for a 25:1 ratio and an 80% encapsulation for a 50:1 ratio. This parameter shows a slight increase for the formulation which has a higher proportion of polymer (50:1), although both show a good encapsulation efficiency as demonstrated in agarose gel retardation assay qualitatively (Figure 1C.1).

Additionally, these nanoparticles were transfected in the melanoma cell line of B16F10, as can be observed in Figure 1G qualitatively, where a percentage of 73.4 (25:1) and 71.6% (50:1) of GFP-positive cells (Figure 1D,E) revealed that our formulated nanoparticles had been able to deliver the genetic material properly to finally be expressed by cells. The cytotoxicity assay (Figure 1F) revealed a cell viability of 86 (25:1) and 67% (50:1).

#### Formation and Release of the Multilayered Structure of Coated Microneedles

Besides the capacity of the new Bor-pBAE to perform nanoparticle polyplexes to deliver nucleic acids, the next step was related to engineering a coating of microneedles based on a PEM assembly. Bor-pBAE nanoparticles have been used in the preliminary experiments as a control to observe the capability of our coating to form these polyplexes resembling nanoparticles, deeming them the ideal vehicle for DNA transfection. In the following, they are used to form the multilayered polyelectrolyte system, where they are expected to allow the control of the release of each layer, forming polyplexes under pH = 7.4 and pH = 5.1. Once the coating starts its release, the layers will begin to detach from the surface in a generalized manner, releasing their components and forming polyplexes previously analyzed.

In this study, metallic MNs were selected based on their complete insertion, thanks to their high stiffness and yield strength. In the literature, this approach has been already used in breast cancer vaccination, delivering siRNA, or treating eye diseases by intracorneal administration.^43−45^

As the first layer, before the addition of the iterative Bor-pBAE polymer and nucleic acid layers, it was necessary to functionalize the surface of the microneedles with glucose. As described in the experimental section, a thin layer of pp-PFM was incorporated. This procedure has been previously reported by our group as a straightforward manner to immobilize biomolecules on surfaces for biomedical applications.^30,32,46^ pp-PFM polymers can immobilize amine-containing biomolecules, thanks to the high electrophilic character of the perfluorinated ester function that assists a rapid amide formation (see Figure S31, from SI).

Next, the coated substrates were incubated with glucosamine to induce sugar immobilization in the backbone of pp-PFM. Then, the incorporation of the first layer of the borylated poly(β-aminoester) is possible. As mentioned above, the boronic acids present in the Bor-pBAE polymer can interact with the free hydroxyl groups of glucose at pH = 8. Following, a layer of model nucleic acid of choice (concretely pGFP) was deposited over the Bor-pBAE. Based on this process, up to five consecutive layers of Bor-pBAE and GFP plasmid were coated (see the scheme in Figure S32).

**Figure 2:**
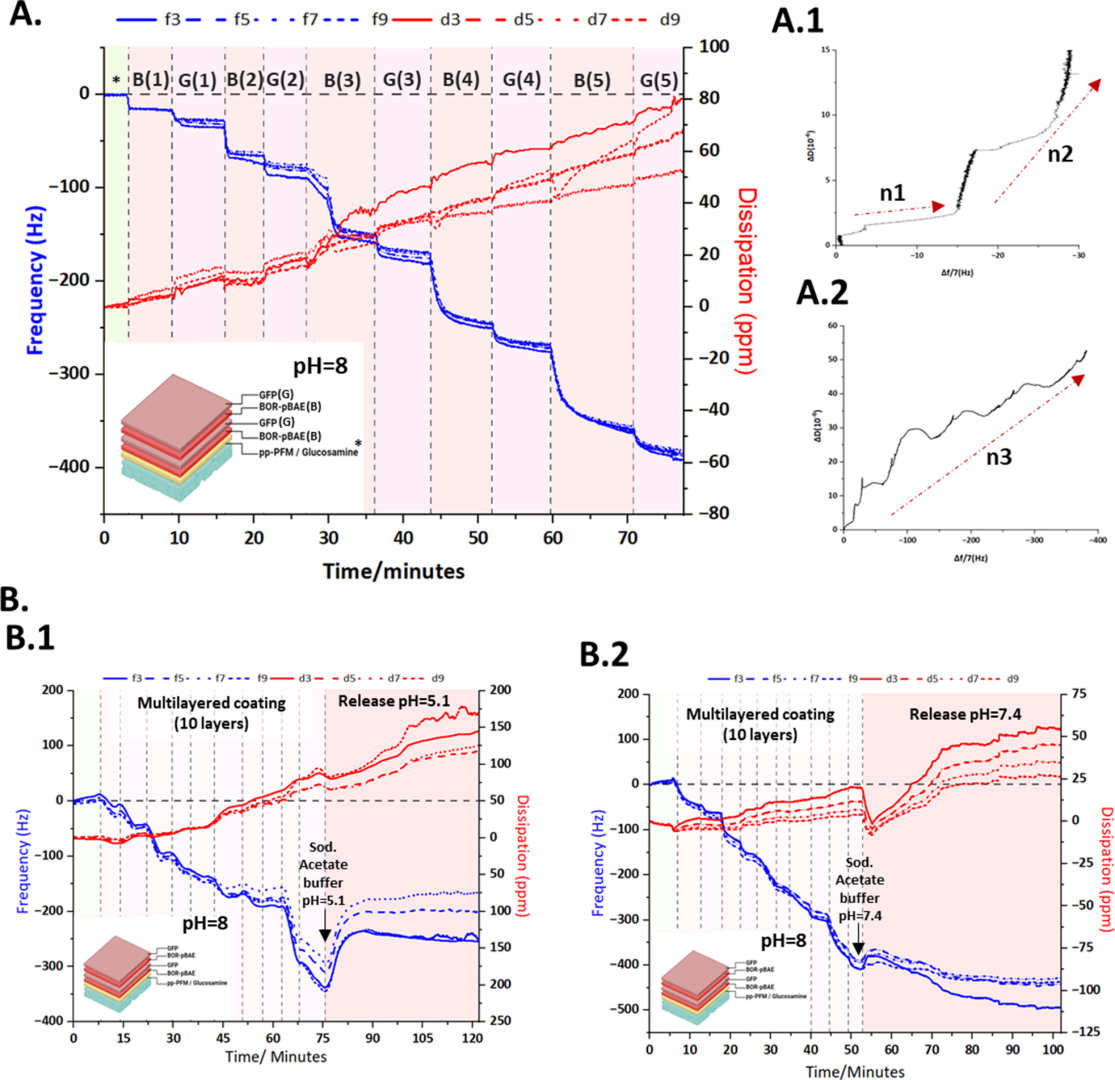
Analysis of PEM multilayer coating formation and release using QCM-D. (A) QCM-D frequency and dissipation profile against time during the formation of a 10-layered PEM system on a pp-PFM/glucosamine-modified sensor. (A.1, A.2) Representative QCM-D7th overtone Δ*D*–Δ*f* plots for addition of Bor-pBAE (arrow n1)/pGFP (arrow n2) layers on a pp-PFM/glucosamine-modified gold sensor (A.1) and addition of all layers of the PEM system (A.2). (B) Study of the release performed after the formation of the PEM system by Q-CMD at pH = 5.1 (B.1) and pH = 7.4 (B.2). (B) Bor-pBAE layer; (G) GFP layer, *pp-PFM/glucosamine-covered sensor.

The formation of the multilayered polyelectrolyte structure of the coated microneedles was confirmed by QCM-D (Figure 2A,B).

Quartz crystal microbalance–dissipation (QCM-D) is a well-suited methodology to characterize small mass changes on the surface of the sensors, which can be related to significant frequency shifts. Changes in the dissipation signal over time are related to variations on the viscoelasticity of the surface of the sensor^46,47^ that together with the frequency profile provides insights into the conformation of the PEM assembly. Thereby, a 10-layered PEM system (Bor-pBAE/pGFP, five times each deposited alternatively) was immobilized on a pp-PFM/glucosamine-modified QCM-D gold sensor. The modification of the sensors allows us to study how the different layers interact with the glucose monolayer covalently attached to pp-PFM due to its affinity with amine groups, as previously described in refs (46 and 47). Besides that, it is possible to mimetize the release of the multilayered coating by QCM-D after performing the deposition of the PEM system. In our experiment, we studied the pH dependence of the interactions of borylated moieties present in our Bor-pBAE. When the multilayered coating is performed in sodium acetate buffer pH = 8 (Figure 2A), the interaction between the diols of the glucose and boronic moieties is favored. On the contrary, a release is expected if the pH is changed. Two pHs were studied: pH= 5.1 (representing the pH of the skin) and pH= 7.4 (representing the physiological pH) (Figure 2B.1,B.2).

Figure 2A shows the frequency and dissipation profile during the formation of the 10-layered PEM system. Initially, a baseline was obtained with sodium acetate buffer pH = 8 to stabilize the glucose monolayer on the sensors’ surface. Then, the Bor-pBAE solution at min 4 encounters the modified sensor observing the first frequency shift of 45 Hz after signal stabilization, indicating its immobilization on the surface of the glucose monolayer demonstrating the good reproducibility of the interaction between the boronic acid of the Bor-pBAE and sugar diols.

Once the Bor-pBAE layer was immobilized on the surface, the pGFP solution was pumped through the QCM-D chamber to promote the formation of the second layer of the PEM system. A frequency shift indicating an increase of the surface mass due to the interaction of pGFP was observed, and when the frequency signal was stabilized, the plateau of the profile showed an increase of 30 Hz.

A Sauerbrey model was used to provide insights into the thickness increase in each PEM layer (Figure S33, from SI) observing that the thickness of the first Bor-pBAE layer was around 10 nm and that of the pGFP layer was around 5 nm.

Interestingly, when the Bor-pBAE solution interacts with the pGFP layer, the frequency shift is more acute (110 Hz, corresponding to 18 nm). The mechanism of the interaction between pGFP and the Bor-pBAE polymer is probably a mixed contribution of electrostatic interaction between the protonated arginine of the polymer and negatively charged pGFP, and some sort of interactions propitiated by the boronic acid function could be hypothesized.^48^

From this point, frequency shifts occur whenever either Bor-pBAE or pGFP is pumped through the chamber to be homogeneously distributed, demonstrating the interactions between layers by increasing the mass over the modified sensor. Additionally, slight dissipation shifts are registered, but during the assembly of the layers, this parameter remains almost invariable (Figure 2A).

Δ*f* and Δ*D* signals were obtained from QCM-D measurements, observing different patterns when representing ΔD versus Δf shifts among the formation of the different layers (Figure 2A.1,A.2). Figure 2A.1 shows the formation of the first layers of Bor-pBAE/pGFP. In this graph, the frequency and the dissipation increase at each layer formed, showing a first increase coming from the polymer (Figure 2A.1 arrow n1) and a deeper increase when pGFP is added (Figure 2A.1 arrow n2). As shown in Figure 2A.2 (arrow n3), the tendency of Δ*f* and Δ*D* increases during the formation of the whole PEM system, which means that there is a general homogeneous deposition of all 10 layers of polymer and pGFP. This coating model leads to a well-established multicoating system of layers of Bor-pBAE and pGFP.

Once the electrolyte multilayered coating is finished, we would like to check the capacity of reversibility of the borylated moieties of the Bor-pBAE (taking into account the p*K*_a_) under different pH conditions, as commented before.

As shown in Figure 2B.1, the pH change starts at min 75 (pH = 5.1). A change in frequency is observed, indicating the release of deposited layers, as we have hypothesized. Regarding dissipation, it is noted that when the release starts, a slight increase is observed, and then, it is again stabilized and increases progressively indicating a very small change in the viscoelasticity of the construct deposited.

In Figure 2B.2, pH= 7.4 (reproducing physiological conditions) is studied. In this change, the frequency changes indicate a modification in the interaction between the layers, with a subsequent release, but less intense than at pH 5.

Taken altogether, it seems that part of the multilayered coating is released in a sustained manner. At pH = 5.1, the delivery starts faster during the first few minutes of the release compared to pH = 7.4. The acidic pH = 5.1, below the p*K*_a_ of the Bor-pBAE (p*K*_a_ = 6.8), promotes the disassembly of ester complexes between boronic moieties of Bor-pBAE and glucose diols of immobilized glucosamine.^26,40^

The QCMD technique allows us to also study the changes in the viscoelasticity of the PEM system. The dissipation profile in Figure 2 indicates that during the formation of the different layers of the PEM system, no significant changes in viscoelasticity occurred. This may indicate that the layers formed have a similar composition, and their viscoelasticity (led by the film's ability to absorb water) is mediated by the presence of the Bor-pBAE polymeric layer.

#### Composition of the Release of the Multilayered Coated Microneedles

From this point, a multilayered coating was performed over the metallic surface of microneedles, and the stability of the layers could be tuned, thanks to a pH change. The question is to know if the release of the layers leads to the formation of polyplex nanoparticles that are able to transfect the surrounding cells.

**Figure 3:**
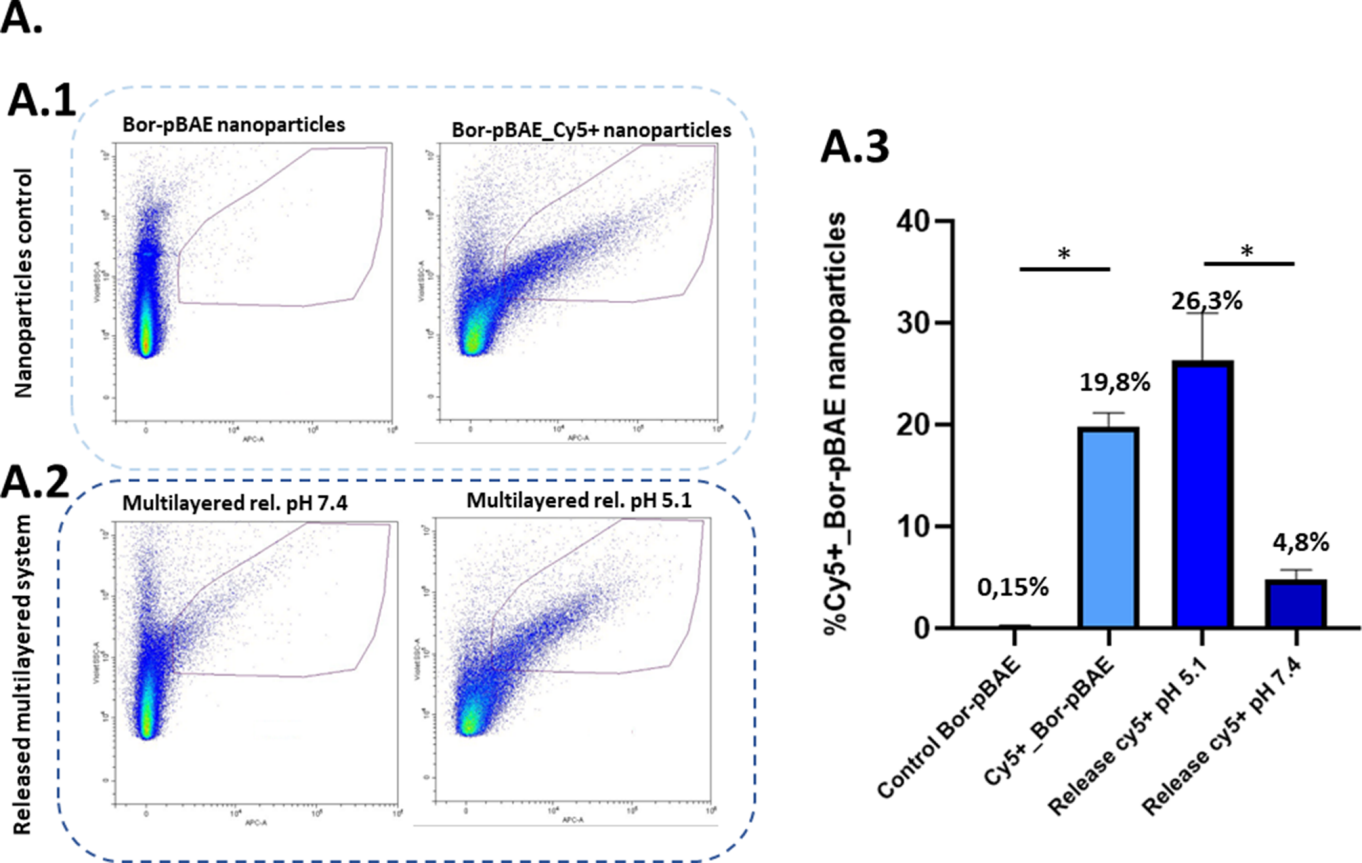
Flow cytometry assay of tracking Cy5-positive Bor-pBAE nanoparticles. (A) Analysis of the PEM release using flow cytometry: control of nanoprecipitated Bor-pBAE nanoparticles without Cy5 (left) and with Cy-5 (right) (A.1); PEM release of Cy-5-positive Bor-pBAE nanoparticles at pH = 7.4 (left) and pH = 5.1 (right) (A.2). Representation of Cy5-positive BorpBAE nanoparticles (A.3). B: Bor-pBAE, G: pGFP.

In this sense, the experiment performed was based on studying the composition of the release media coming from the multilayered coating of the microneedles after 1 h of incubation under different pH conditions: the physiological pH = 7.4 and the acidic pH = 5.1. To track the Bor-pBAE polymer in the formation of polyplexes, this polymer was labeled by Cyanine 5 fluorophore (from now on Cy-5). Afterward, these released media were analyzed using the Cytoflex S cytometer. To isolate the gating area corresponding to the size of nanoparticles, a commercial reference sample composed of fluorescent nanoparticles of different sizes was used. In Figure S34 graph 1, the positive control of Cy-5 Bor-pBAE nanoparticles is represented. Then, graph 2 shows the representative gated area of Cy5 particles (the ones displaced to the right in graph 1) and a range of the different size regions which had been pre-established by the reference sample of commercial fluorescent nanobeads. As observed in Figure S34 graph 2, the positive Cy5 Bor-pBAE nanoparticles correspond to a range of sizes between 150 and 250 nm. Taking this sample as a positive reference of Cy-5 nanoparticles, the analysis of the released media was carried out. In Figure 3A.2,A.3, it can be observed that both conditions of pH display a release of Cy5-positive Bor-pBAE nanoparticles compared with the Cy-5-positive control of Bor-pBAE nanoparticles (right panel, Figure 3A.1). At pH = 5.1, the Cy5+ Bor-pBAE nanoparticles released show a high number of nanoparticles (26.3%) compared to the ones released at pH = 7.4 (4.8%), as we could expect from the QCM-D results. These values are similar to the ones obtained with the positive control Cy5+ Bor-pBAE nanoparticles (19.8%). Regarding the size of those particles, the released Cy5+ Bor-pBAE particles coming from the pH = 7.4 condition showed a range of sizes between 150 and 300 nm (Figure S35, graph2) whereas Cy5+ Bor-pBAE particles released at pH = 5.1 displayed a range of sizes between 150 and 250 nm (Figure S36, graph 2). In this sense, the acidic pH allows the formation of Cy5+ Bor-pBAE nanoparticles more similar to the positive control of nanoprecipitated Cy5+ Bor-pBAE nanoparticles.

As a reference, the controls applied in this experiment are nonlabeled Bor-pBAE nanoparticles (left panel, Figure 3A.1) and Cy5+ labeled nanoparticles to compare with the experimental groups of released media.

#### Confirmation of the Functionality of the Release of PEM System

Once the multilayered coating was characterized and the release was studied, the final step was related to the interaction of the genetic treatment of pGFP with cellular models after the release.

In this experiment, the multilayered coated microneedles were incubated at 37 °C for 1 h under two different pH conditions (pH = 7.4 and pH = 5.1).

**Figure 4:**
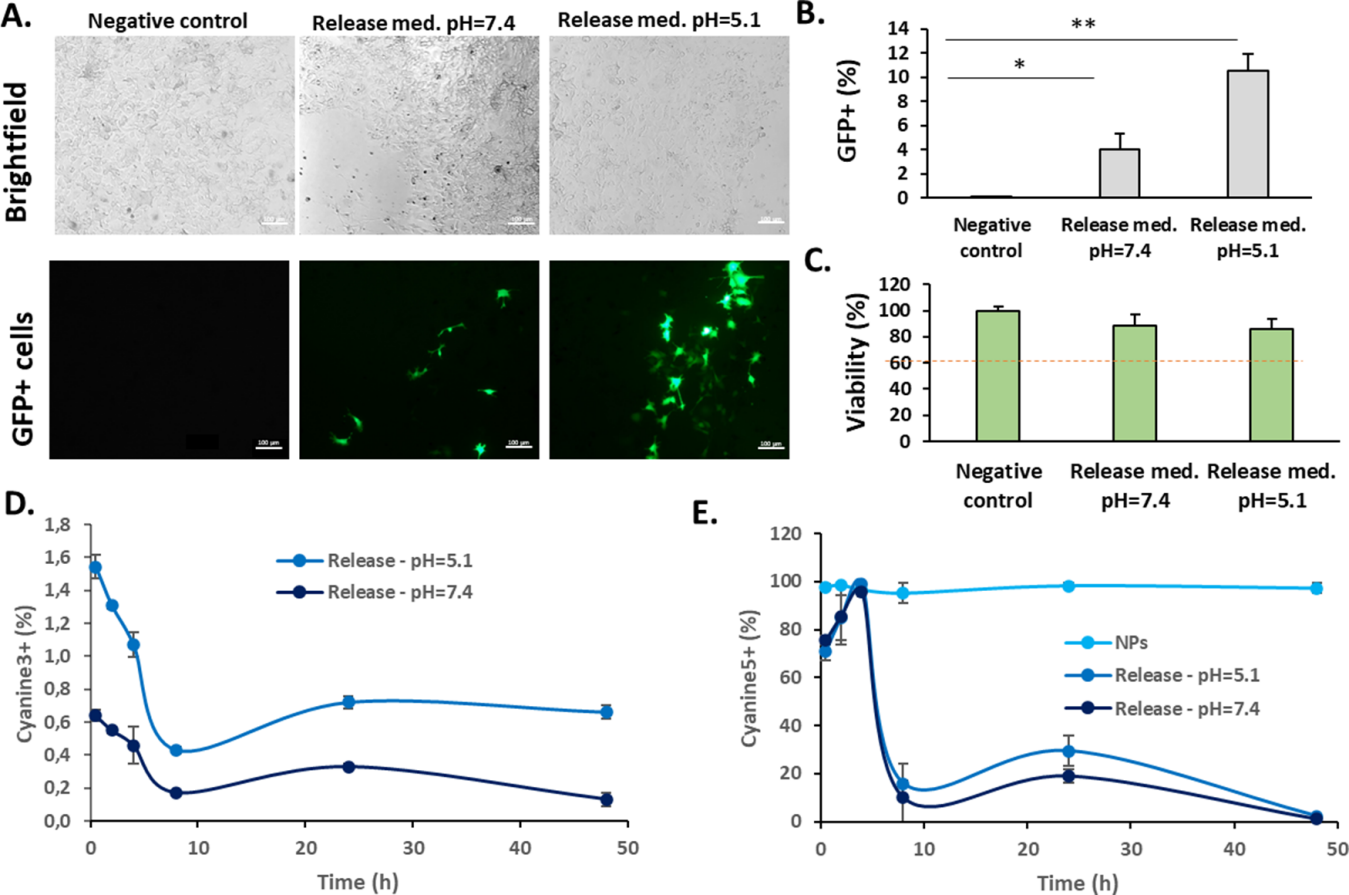
Study of the multilayer release in B16F10 cells. (A) Brightfield and fluorescent images of negative control cells and cells transfected with release media from both conditions of pH (pH = 7.4 and pH = 5.1). (B) Flow cytometry analysis of cells transfected after 48 h. (C) Cell viability analysis at 48 h after transfection. (D) Plasmid uptake (labeled with Cyanine 3). (E) Polymer uptake (labeled with Cyanine 5). Statistical analysis (*p < 0.05; **p < 0.01). Release med.; release medium; pGFP: plasmid green fluorescent protein.

Then, the release media were transfected in B16F10 melanoma cells. Melanoma cells were selected as a simultaneous model of skin and oncological disease, as described in previous similar studies.^22^ Results revealed that the plasmid released from both pH conditions tested was able to successfully transfect model cells (Figure 4A,B) without producing any significant cytotoxicity (Figure 4C). As expected from nanoparticle-formulated pBAEs, and from our previous reports using these polymers, in all tested conditions (see Figures 1F and 4C), viability is above 60% as required by guidelines. Concretely, 4% of transfected cells with a release medium at pH 7.4 and 10.6% of transfected cells with a release medium at pH = 5.1. To highlight that in this experiment, all media released, including all the nanoparticles formed by the plasmid interaction with the polymer, was incubated with the cells. Consequently, the final amount of nucleic acid is probably different between conditions. Nevertheless, the experiment aimed to compare the output in a physiological setup, where the amount of released plasmid will be dependent on the pH and not normalized to other conditions.

To go further on the release kinetics study, the uptake of the plasmid and the polymer, both labeled with fluorophores, from the multilayered microneedles, has been quantified by flow cytometry (Figure 4D,E, and qualitative assessment in Figures S38 and S39, from SI). As expected from the transfection results, the percentages of polymer from nanoparticles are significantly higher than those of the polymer released from the multilayered microneedles. After an initial 4 h lag time, where the polymer from the most external layer is directly uptaken by cells, there is a decrease in the polymer uptake over time, attributed to the decrease in the total polymer amount in the cell media. Concerning the plasmid uptake, it is extremely lower, as compared to the values of the polymer. Again, this was an expected result, due to the impossibility of the plasmid to penetrate the cells, unless it is formulated as nanoparticles with the polymer. On the contrary, the polymer, being cationic, can enter without being nanoformulated, and this is why, these higher uptake percentages are obtained. Finally, and again, as expected, the pH = 5.1 release is higher than that of the pH = 7.4 due to the nature of the pH-sensitive polymer.

To note, although transfection percentages may seem low, these are sufficient to achieve a therapeutic activity and are equivalent to previous results in literature studies. For example, for in vivo mRNA vaccination purposes, Perche et al. achieved around 5% of liposome-coated lipoplex transfection to dendritic cells,^49^ which is equivalent to the present results and also to our previous study^9^ with pBAE polyplexes.

#### Microneedles as Multilayered Coated Devices To Perform Transdermal Delivery

**Figure 5:**
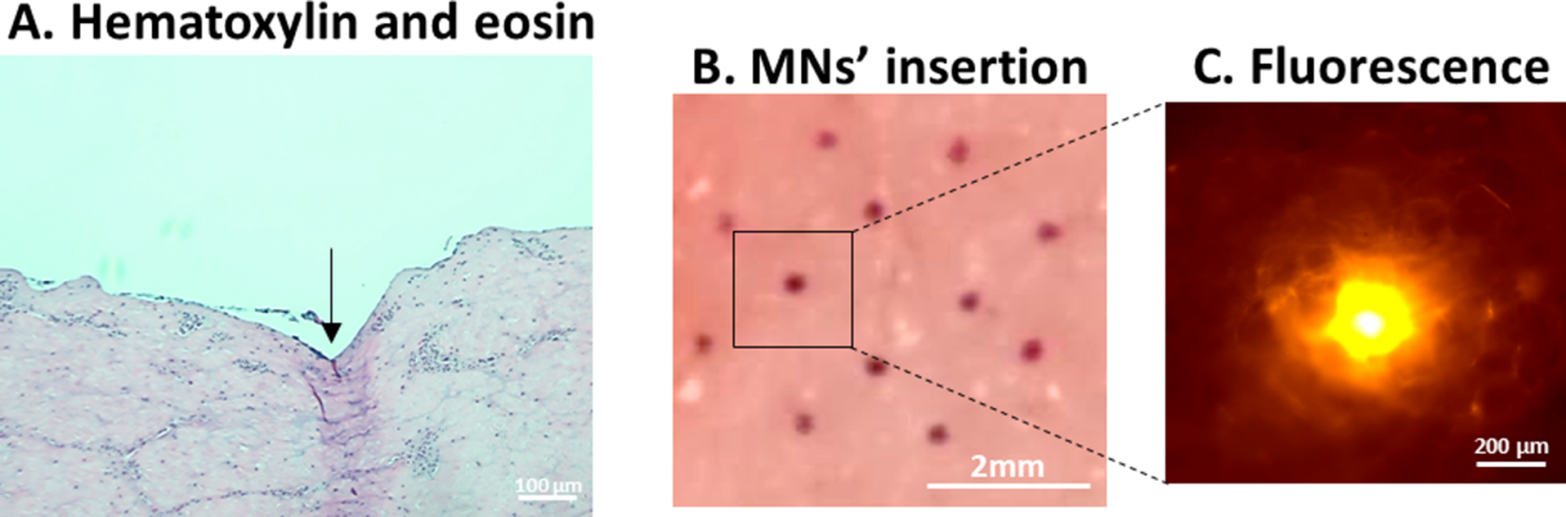
Insertion studies of microneedle devices using ex vivo models of porcine skin**.** (A) Hematoxylin and eosin staining of the profile of the skin which had been inserted by the microneedle devices (the arrow shows the place of insertion). (B) Pink holes performed by the insertion of the microneedle devices coated by the multilayered treatment (regarding the PEM system, pGFP had been conjugated with Cyanine 3 fluorophore). (C) Fluorescence image of Cyanine 3 fluorophore as one of the representative holes performed by the microneedle device.

To finally check if our multilayered coated microneedles were able to distribute the treatment over the skin, we carried out the insertion studies of the devices using cadaveric porcine skin samples as an ex vivo model. In this sense, to confirm that our devices were able to penetrate the first layers of the skin and deliver the treatment, our approach was to perform a transdermal application over porcine skin (Figure 5A,B). To track the distribution of our multilayered treatment, pGFP layers were labeled by Cyanine 3 fluorophore. Thus, the pink holes observed in Figure 5B demonstrate the deposition of the treatment over the first penetrated layers of the skin, and one of those holes has been zoomed up to check the presence of fluorescence coming from the Cy3-labeled pGFP (Figure 5C). The release of the multilayered treatment over the skin is confirmed to be applied as a future transdermal device in a painless and user-friendly way of administration.

## Conclusions

The generation of PEM microneedle platforms for controlled nucleic acid delivery is an appealing option for the painless and patient-friendly treatment of many skin and immune-based diseases. Here, we report for the first time the synthesis and use of a borylated OM-pBAE for the generation of a platform of MNs. The construct is composed of sequential and iterative layers of this newly generated **Bor-pBAE** polymer alternated with DNA. Our system is attached to a metallic surface through their prior functionalization with an initial layer of pp-PFM followed by a layer of glucosamine, with demonstrated affinity to borylate groups. Further, we demonstrated that these MNs can protect the genetic material from being degraded, thanks to the multilayered system, and release a model plasmid DNA inside nanoparticles that are capable of efficiently interacting with skin cells; thus, the use of these MNs for the controlled delivery of nucleic acids is confirmed.

Summarizing, we established an MN platform that may be ultimately applied for the prevention and treatment of unmet medical needs requiring the transdermal controlled administration of nucleic acids.

## Supporting Information Available

Preparation of a model fluorinated boronic acid derivative; characterization of **FPBpin-COOBu** by ^1^H NMR; characterization of **FPBpin-COOBu** by ^13^C NMR; characterization of **FPBpin-COOBu** by ^19^F NMR; characterization of **FPBpin-COOBu** by ^11^B NMR; characterization of **FPBpin-COOBu** by FTIR; characterization of **FPBA-COOBu** by ^1^H NMR; characterization of **FPBA-COOBu** by ^13^C NMR; characterization of **FPBA-COOBu** by ^19^F NMR; characterization of **FPBA-COOBu** by ^11^B NMR; characterization of **FPBA-COOBu** by FTIR; p*K*_a_ determination of **FPBA-COOBu**; characterization of **C6** poly(β-aminoester) by ^1^H NMR; characterization of **C6** poly(β-aminoester) by ^13^C NMR; characterization of **C6-FPBpin** poly(β-aminoester) by ^1^H NMR; characterization of **C6-FPBpin** poly(β-aminoester) by ^13^C NMR; characterization of **C6-FPBpin** poly(β-aminoester) by ^19^F NMR; characterization of **C6-FPBpin** poly(β-aminoester) by ^11^B NMR; characterization of **C6-FPBpin** poly(β-aminoester) by FTIR; characterization of **C6-FPBA** poly(β-aminoester) by ^11^B NMR; characterization of **C6-FPBA** poly(β-aminoester) by ^13^C NMR; characterization of **C6-FPBA** poly(β-aminoester) by ^19^F NMR; characterization of **C6-FPBA** poly(β-aminoester) by ^11^B NMR; characterization of **C6-FPBA** poly(β-aminoester) by FTIR; ^1^H NMR control spectra (in DMSO-*d*_*6*_) of the diacrylate-terminated poly(β-aminoester) **C6** upon treatment with TFA; characterization of **C6-FPBA-C**_**2**_**R**_**6**_ (**Bor-pBAE**) by ^1^H NMR; characterization of **C6-FPBA-C**_**2**_**R**_**6**_ (**Bor-pBAE**) by ^13^C NMR; characterization of **C6-FPBA-C**_**2**_**R**_**6**_ (**Bor-pBAE)** by ^19^F NMR; characterization of **C6-FPBA-C**_**2**_**R**_**6**_ (**Bor-pBAE**) by ^11^B NMR; characterization of **C6-FPBA-C**_**2**_**R**_**6**_ (**Bor-pBAE**) by FTIR; scheme of the nucleophilic substitution reaction between the active ester of PFM and amine of glucosamine; scheme of the multilayered coating over the microneedle devices; thickness of the 10-layered PEM system coating pp-PFM/glucosamine sensor; establishment of the range of sizes of Cy5+ Bor-pBAE nanoparticles; establishment of the range of sizes of Cy5+ Bor-pBAE nanoparticles released at pH= 7.4; establishment of the range of sizes of Cy5+ Bor-pBAE nanoparticles released at pH= 5.1; confocal micrographs of NP uptake; confocal micrographs of microneedles at pH = 5.1; and confocal micrographs of microneedles at pH = 7.4.
