# Supplementary material for: Designing Polyelectrolyte Microneedles Based on Borylated Poly(β-aminoester) Polymers To Enhance Transdermal pH-Controlled Delivery of Nucleic Acids
Source: ACS Appl Polym Mater. 2024 Jul 24;6(15):8842–55. doi: 10.1021/acsapm.4c00969 (PMC11320383; doi:10.1021/acsapm.4c00969)
Supplement: Supplementary file 1 — ap4c00969_si_001.pdf [file ap4c00969_si_001.pdf]

SUPPORTING INFORMATION

Designing poly-electrolyte microneedles based on borylated poly(β-aminoester) polymers to enhance transdermal delivery of nucleic acids

Patricia González-Sáenz[a,1], Raúl Cosialls[b,1], Robert Texidó[a], Aurora Dols-Perez[c], Ana Belén Cuenca[b], Salvador Borrós[a,*], Cristina Fornaguera[a,*]

[a] Grup d'Enginyeria de Materials (GEMAT, Insititut Químic de Sarrià (IQS), Universitat Ramon Llull (URL), Via Augusta 390, 08017, Barcelona, Spain.

[b] BISI-Bonds/CRISOL group. Dept. of Organic and Pharmaceutical Chemistry, Insititut Químic de Sarrià (IQS), Universitat Ramon Llull (URL), Via Augusta 390, 08017, Barcelona, Spain.

[c] Institut de Bioenginyeria de Cataluña (IBEC), The Barcelona Institute of Science and Technology (BIST), C/ Baldiri I Reixac 11-15, 08028, Barcelona, Spain.

[1] PGS and RC contributed equally to this work.

*Corresponding authors:

- **Salvador Borrós:** *Grup d'Enginyeria de Materials(GEMAT), Institut Químic de Sarrià, Universitat RamonLlull, Barcelona 08017, Spain;* orcid.org/0000-0002-4003-0381; Email: salvador.borros@iqs.url.edu

- **Cristina Fornaguera:** Grup d'Enginyeria de Materials(GEMAT), Institut Químic de Sarrià, Universitat RamonLlull, Barcelona 08017, Spain; orcid.org/0000-0002-7014-3213; Email:cristina.fornaguera@iqs.url.edu

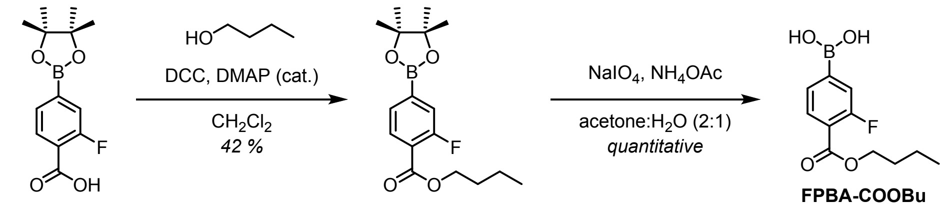

Figure S1. Preparation of a model fluorinated boronic acid derivative. Synthetic sequence towards the preparation of (4-(butoxycarbonyl)-3-fluorophenyl)boronic acid (**FPBA-COOBu**).

<u>Synthesis of **FPBpin-COOBu**:</u> In a 50 mL round-bottom flask, and under argon atmosphere, *n*-butanol (172 µL, 1.9 mmol, 1 equiv.) and the fluorinated aryl boronate derivative **FPBpin-COOH** (500 mg, 1.9 mmol, 1 equiv.) were dissolved in anhydrous CH_2Cl_2 (20 mL) in the presence of DCC (407 mg, 2.0 mmol, 1.05 equiv.) and DMAP (11 mg, 0.09 mmol, 0.05 equiv.). The mixture was then stirred at room temperature for 19 h. The obtained suspension was filtered and stored overnight at 4 °C to force precipitation of the residual traces of dicyclohexylurea. The mixture was again filtered over a 0.45 µm nylon filter and the filtrated was concentrated to dryness. The product was isolated by flash-chromatography eluting with 1:0 to 9:1 cyclohexane:AcOEt gradient mixture (R_f = 0.4 in 9:1 cyclohexane:AcOEt). Colourless oil, 252 mg, 42 % isolated yield.

^{1}H NMR ($CHCl_3$-d_1, 400 MHz) δ 7.89 (dd, $^3J_{H-H}$ = 7.6, $^4J_{H-F}$ 7.0 Hz, 1H), 7.59 (dd, $^3J_{H-H}$ = 7.7, 1.0 Hz, 1H), 7.53 (dd, $^3J_{H-F}$ = 11.1, 0.7 Hz, 1H), 4.34 (t, J = 6.6 Hz, 2H), 1.81 – 1.69 (m, 2H), 1.53 – 1.41 (m, 2H), 1.35 (s, 12H), 0.97 (t, J = 7.4 Hz, 3H).

^{13}C NMR (CHCl$_3$-d_1, 100 MHz) δ 164.8 (J_{C-F} = 3.5 Hz), 164.7 (J_{C-F} = 3.7 Hz), 161.5 ($^1J_{C-F}$ = 260.2 Hz), 131.4, 130.0, 122.8 ($^2J_{C-F}$ = 20.9 Hz), 84.6, 65.4, 30.8, 25.0, 19.3, 13.9.

^{19}F NMR (CHCl$_3$-d_1, 376 MHz) δ -111.42 ($^3J_{F-H}$ = 11.5 Hz, $^4J_{F-H}$ = 7.2 Hz).

^{11}B NMR (CHCl$_3$-d_1, 128 MHz) δ 30.5.

FTIR (ATR) cm^{-1}**:** 2961.3, 1717.4 (C=O st), 1503.3, 1406.9, 1357.7, 1281.5, 1210.2, 1138.8, 1094.5, 965.2, 851.5, 706.8.

HRMS-ESI: calc. for $C_{17}H_{25}BFO_4$ [M+H]$^+$: m/z = 323.1824; found 323.1814.

Synthesis of **FPBA-COOBu**: In a 25 mL round-bottom flask **FPBpin-COOBu** (200 mg, 0.62 mmol, 1 equiv.), $NaIO_4$ (797 mg, 3.7 mmol, 6 equiv.) and NH_4OAc (287 mg, 3.7 mmol, 6 equiv.) were mixed with 22 mL of a solution of acetone:water (2:1). The suspension was allowed to stir at room temperature for 22 h. The mixture was filtered over celite and washed with acetone. The filtrate was evaporated, and the solid residue was washed with pentane (7 mL x 3). The obtained solid was resuspended in MeOH (4 mL), filtered and the filtrate was concentrated to dryness. The product was obtained as a white solid (150 mg) in quantitative yield.

^{1}H NMR (MeOH-d_4, 400 MHz) δ 7.76 (dd, J = 7.4, 7.4 Hz, 1H), 7.43 (d, J = 7.7 Hz, 1H), 7.34 (d, J = 12.3 Hz, 1H), 4.31 (t, J = 6.5 Hz, 2H), 1.79 – 1.69 (m, 2H), 1.57 – 1.43 (m, 2H), 0.99 (t, J = 7.4 Hz, 3H).

^{13}C NMR (MeOH-d_4, 100 MHz) δ 177.0, 166.7, 162.8 (d, $^1J_{C-F}$ = 258.2 Hz), 131.2, 129.8 (d, J_{C-F} = 3.3 Hz), 121.9 (d, $^2J_{C-F}$ = 19.3 Hz), 65.9, 31.9, 20.3, 14.0.

^{19}F NMR (MeOH-d_4, 376 MHz) δ -115.0.

^{11}B NMR (CHCl$_3$-d_1, 128 MHz) δ 29.6.

FTIR (ATR) cm^{-1}**:** 3272.7 (OH st), 2960.3, 1708.7 (C=O st), 1401.1, 1269.0, 1211.1, 1084.8, 935.3, 717.4.

HRMS-ESI: calc. for $C_{11}H_{13}BFO_4$ [M-H]$^-$: m/z = 239.0896; found 239.0893.

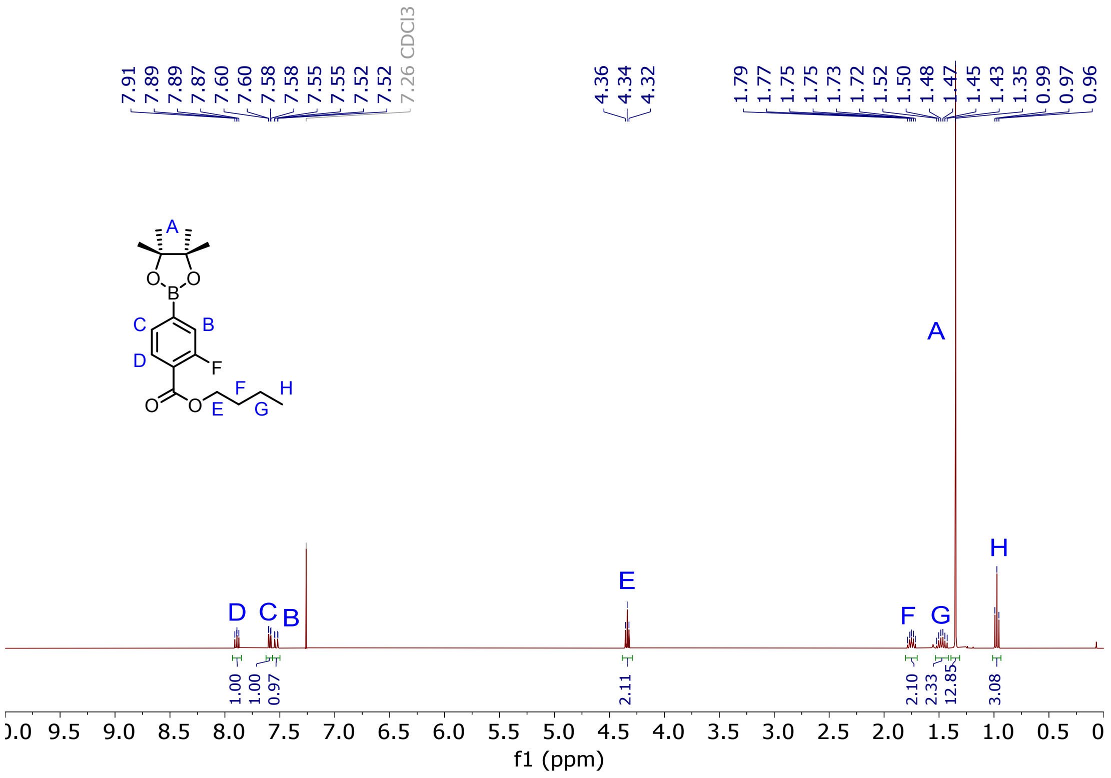

Figure S2. Spectroscopic characterization of **FPBpin-COOBu**: ^{1}H-NMR in $CHCl_3$-d_1.

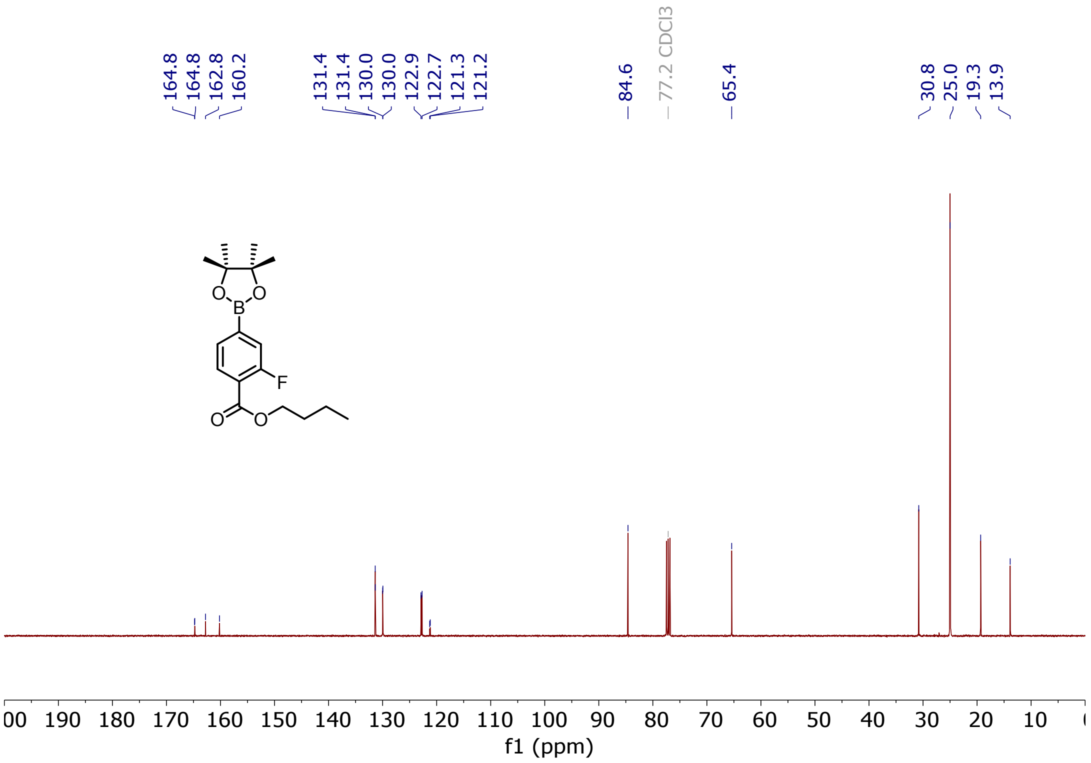

Figure S3. Spectroscopic characterization of **FPBpin-COOBu**: ^{13}C-NMR in $CHCl_3$-d_1.

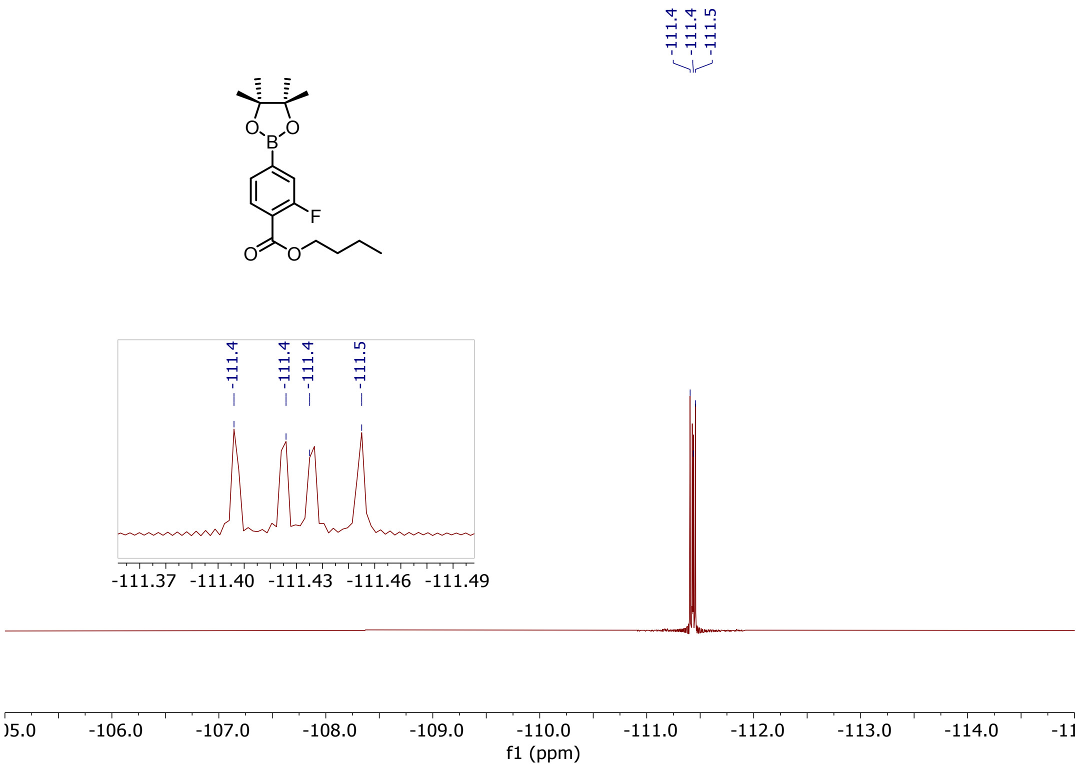

Figure S4. Spectroscopic characterization of **FPBpin-COOBu**: ^{19}F-NMR in $CHCl_3$-d_1.

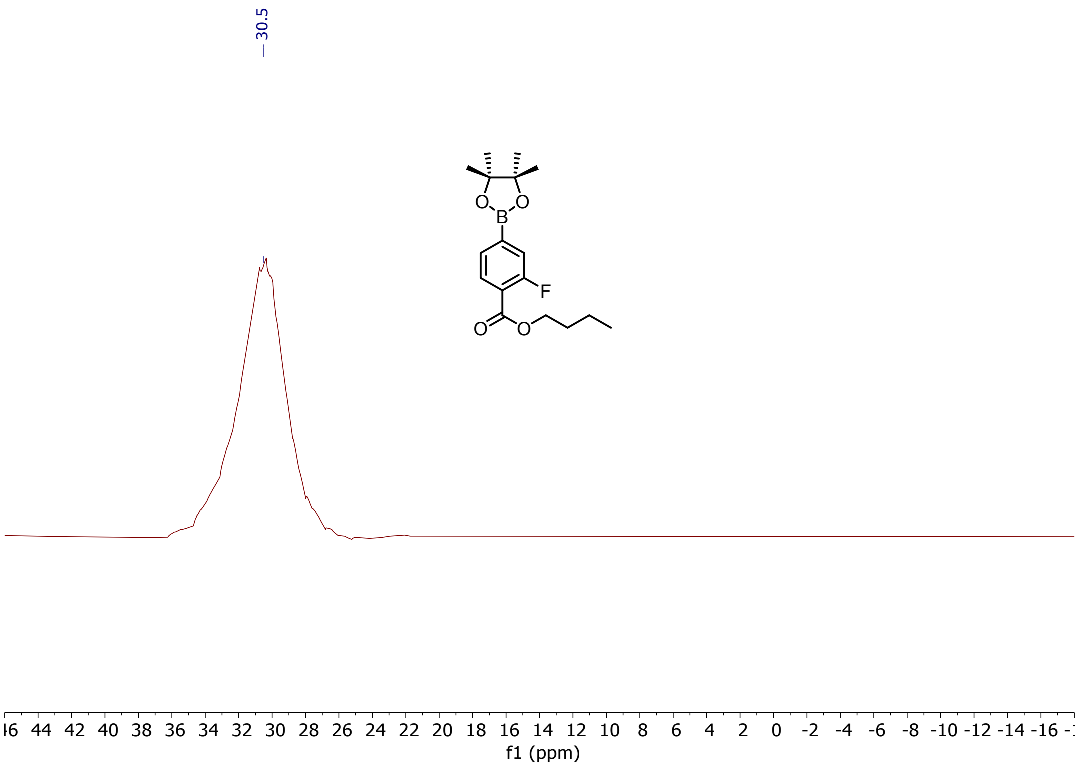

Figure S5. Spectroscopic characterization of **FPBpin-COOBu**: ^{11}B-NMR in $CHCl_3$-d_1.

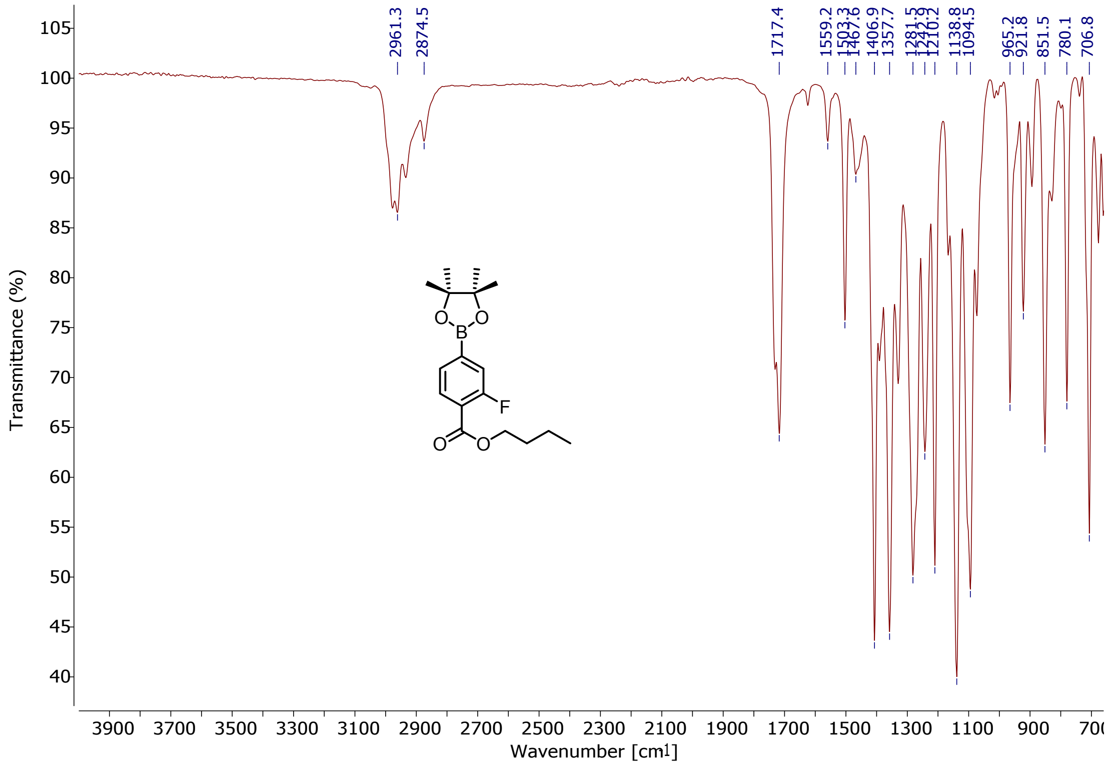

Figure S6. Spectroscopic characterization of **FPBpin-COOBu**: FTIR by ATR (neat).

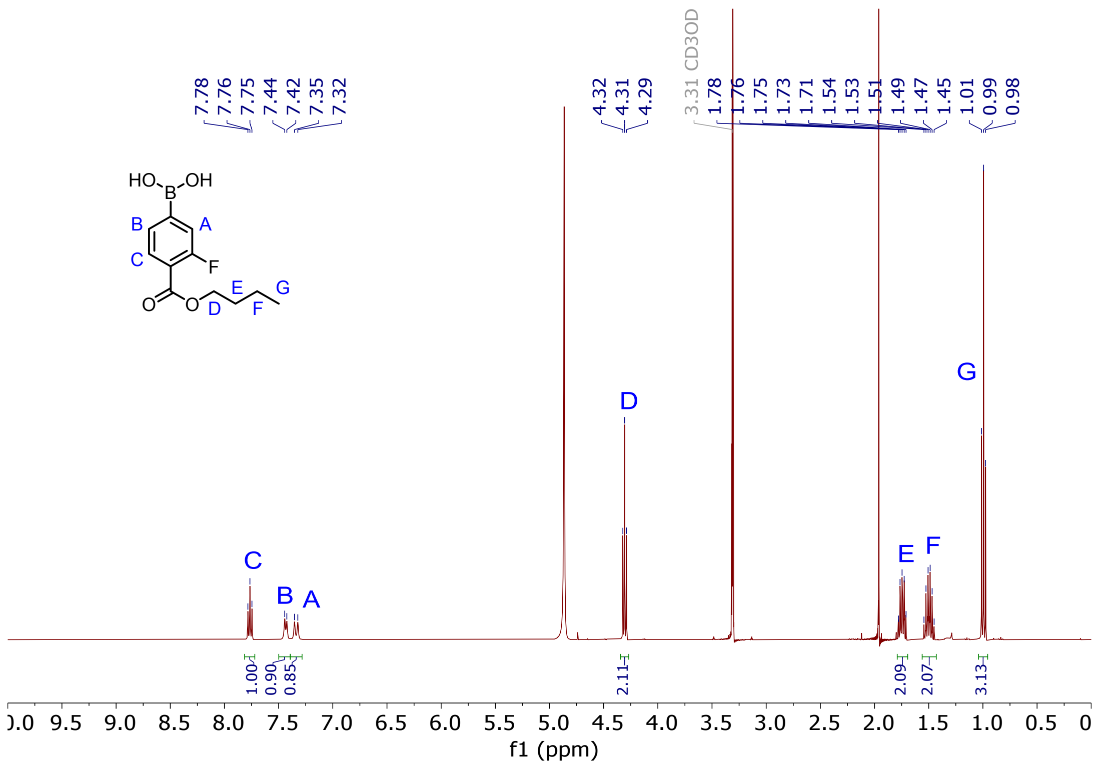

Figure S7. Spectroscopic characterization of **FPBA-COOBu**: ^{1}H-NMR in CH_3OH-d_4.

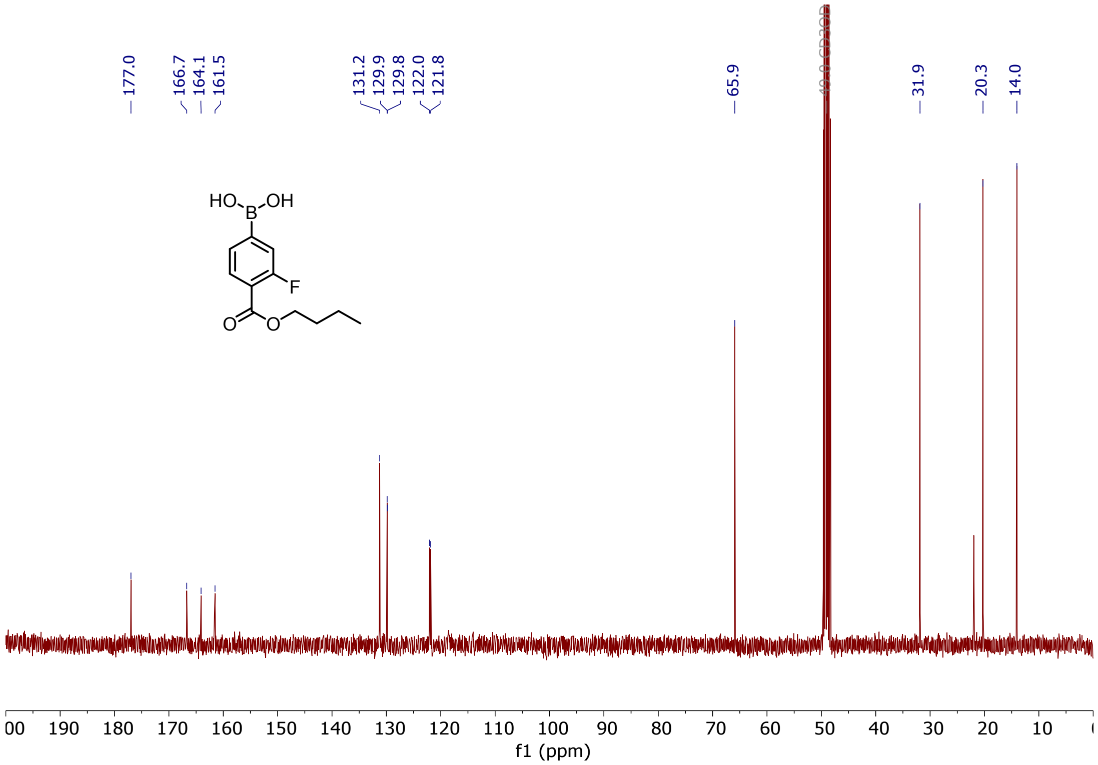

Figure S8. Spectroscopic characterization of **FPBA-COOBu**: ^{13}C-NMR in CH_3OH-d_4.

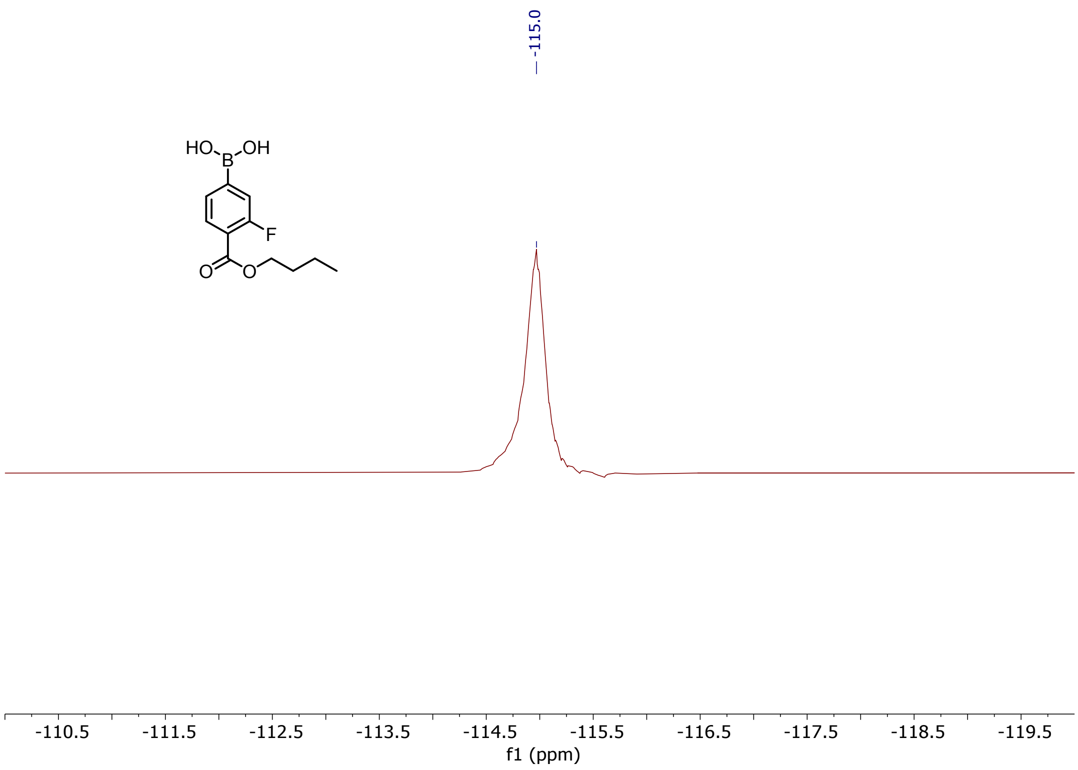

Figure S9. Spectroscopic characterization of **FPBA-COOBu**: ^{19}F-NMR in CH_3OH-d_4.

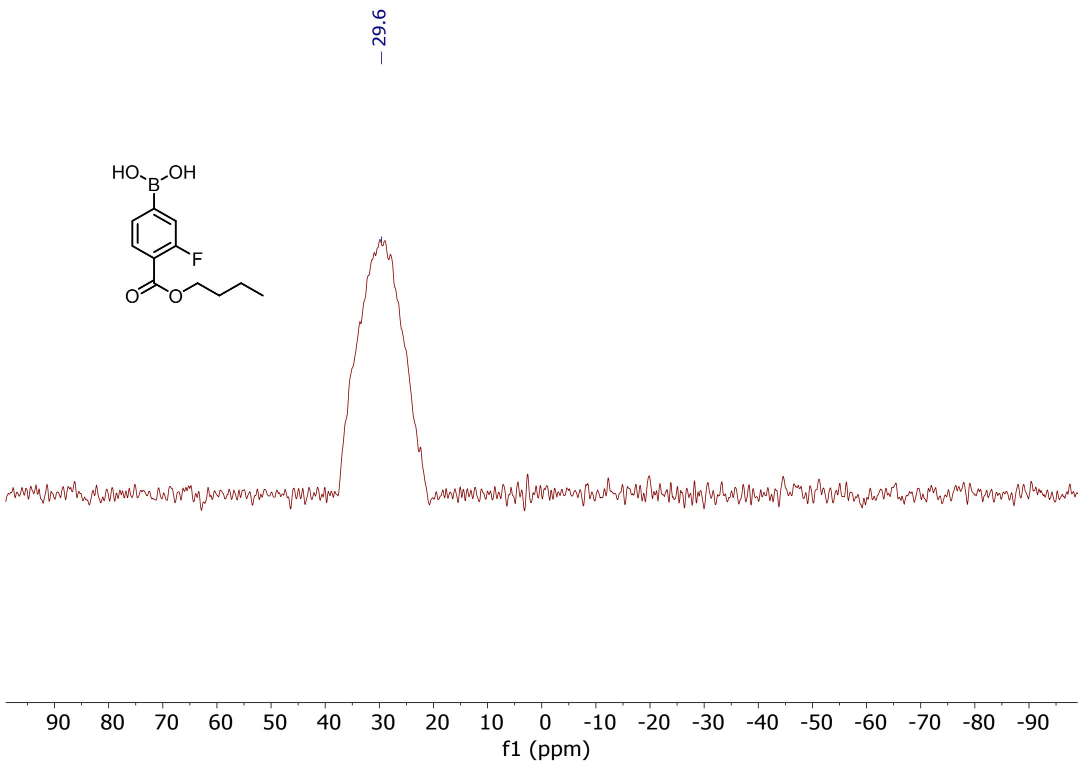

Figure S10. Spectroscopic characterization of **FPBA-COOBu**: ^{11}B-NMR in $CHCl_3$-d_1.

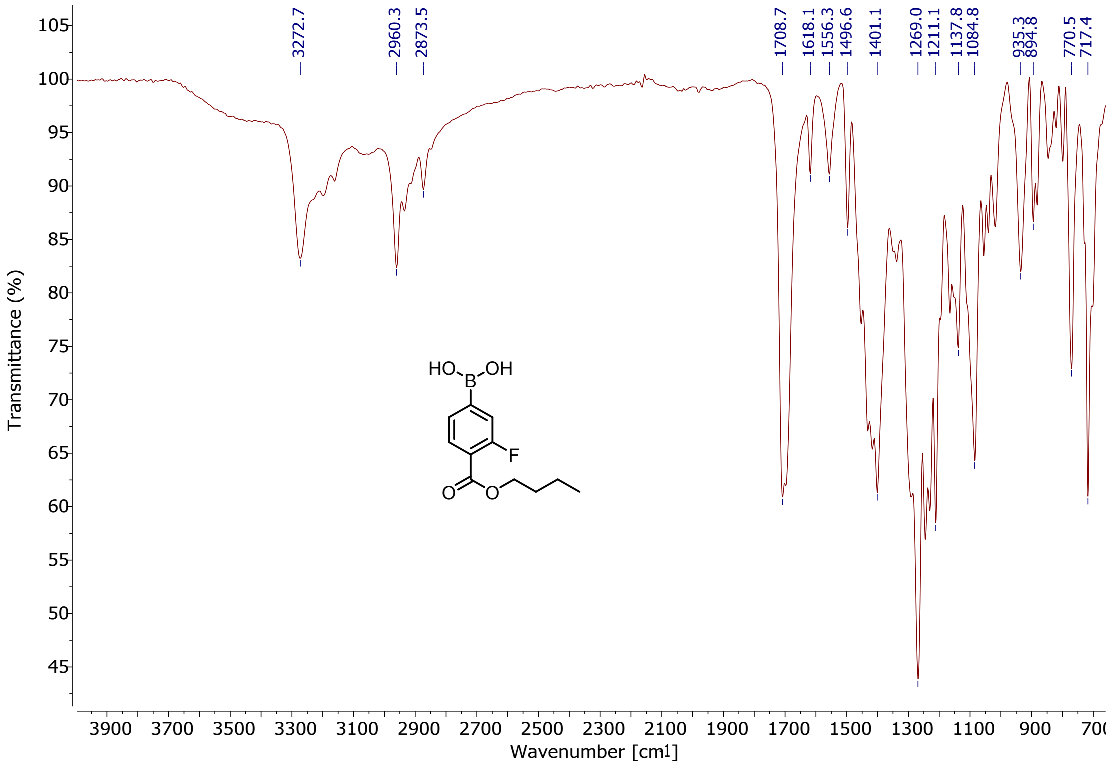

Figure S11. Spectroscopic characterization of **FPBA-COOBu**: FTIR by ATR (neat).

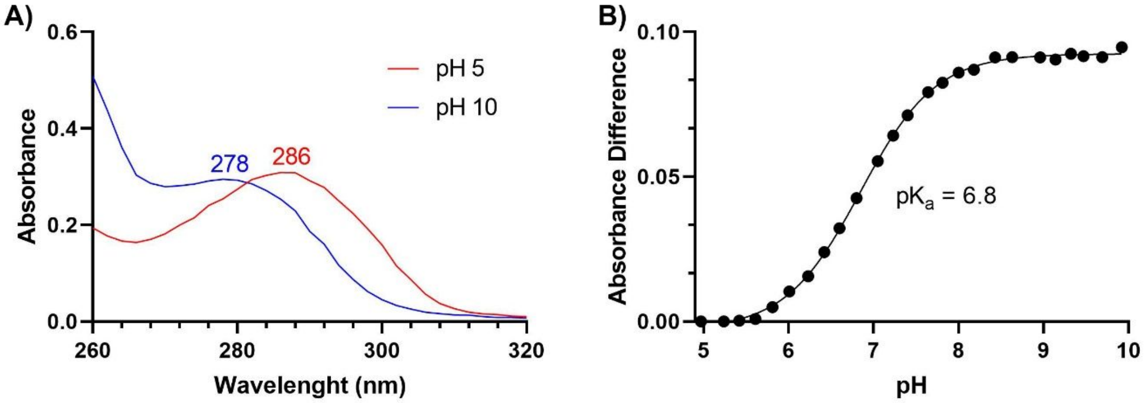

Figure S12. pK_a determination of **FPBA-COOBu**. A) Maximum absorbance bands of **FPBA-COOBu** at pH 5 and pH 10. B) Plot of the absolute difference between the absorbance at 278 and 286 nm at different pH to determine the apparent pK_a.

The pK_a of the esterified FPBA model was determined spectroscopically measuring in a quartz cuvette at multiple pH the difference between the absorbance at 278 (tetravalent form) and 286 nm (trivalent form) of a 0.2 mM **FPBA-COOBu** solution in aqueous sodium phosphate buffer solution (0.1 M) in the presence of 2% DMSO. The absolute absorbance difference was plotted as a function of pH and fitted to a four-parameter logistic curve. The apparent pK_a was calculated as the inflexion point of the curve.

^{1}H NMR (DMSO-d_6, 400 MHz) δ 6.31 (d, *J* = 17.3 Hz, 2H), 6.21 – 6.10 (m, 2H), 5.93 (d, *J* = 10.3 Hz, 2H), 4.11 (t, *J* = 5.9 Hz, 4H), 4.08 – 3.91 (m, 24H), 3.36 (t, *J* = 6.6 Hz, 6H), 2.64 (t, *J* = 6.9 Hz, 24H), 2.43 – 2.24 (m, 36H), 1.71 – 1.50 (m, 29H), 1.49 – 1.12 (m, 44H), 0.84 (t, *J* = 6.7 Hz, 10H).

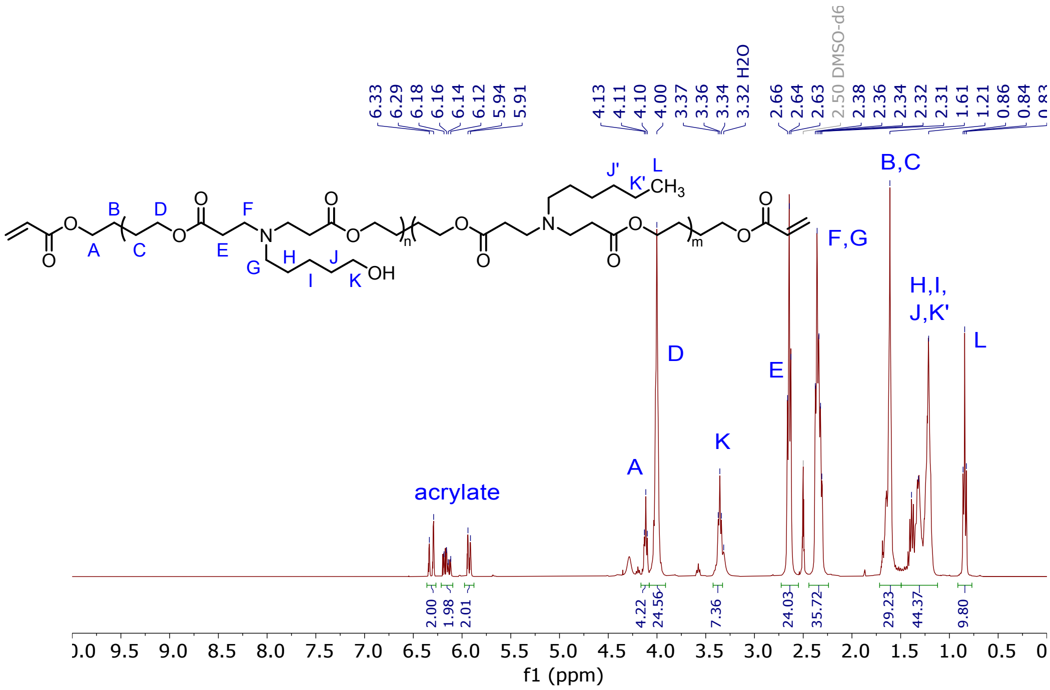

Figure S13. Spectroscopic characterization of **C6** poly(β-aminoester): ^{1}H-NMR in DMSO-d_6.

^{13}C NMR ($CHCl_3$-d_1, 100 MHz) δ 172.7 (O=$C_{\beta\text{-aminoester}}$), 166.2 (O=$C_{acrylate}$), 130.8, 128.4, 64.0, 63.9, 62.4, 53.6, 49.2, 32.5, 31.7, 27.0, 26.8, 25.3, 23.4, 22.6, 14.1.

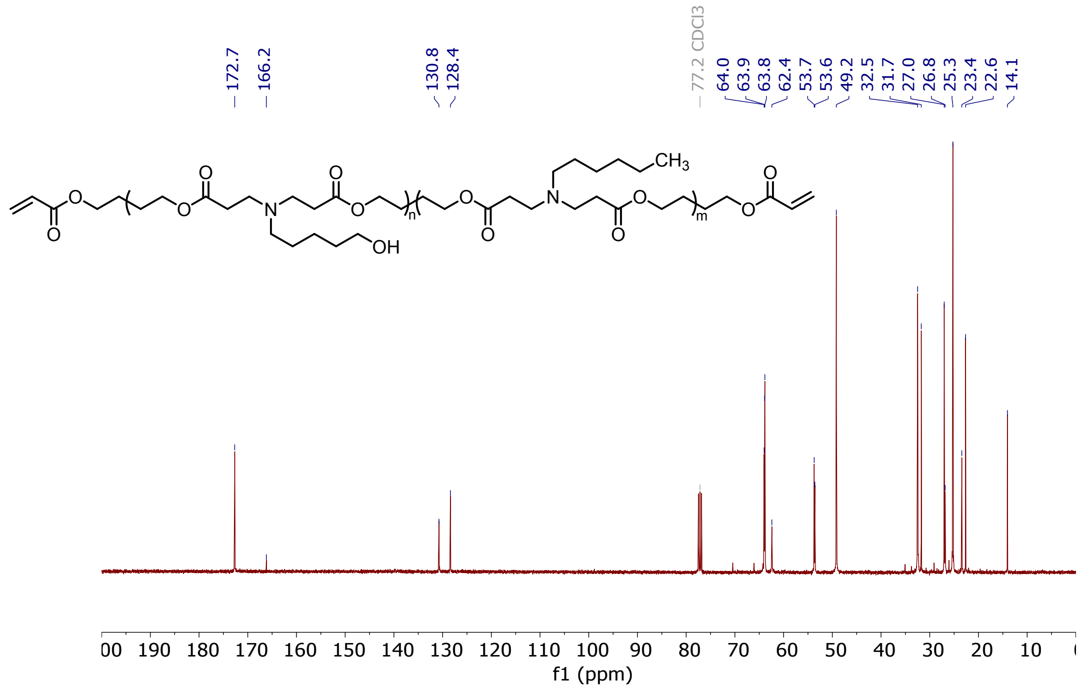

Figure S14. Spectroscopic characterization of **C6** poly(β-aminoester): ^{13}C-NMR in $CHCl_3$-d_1.

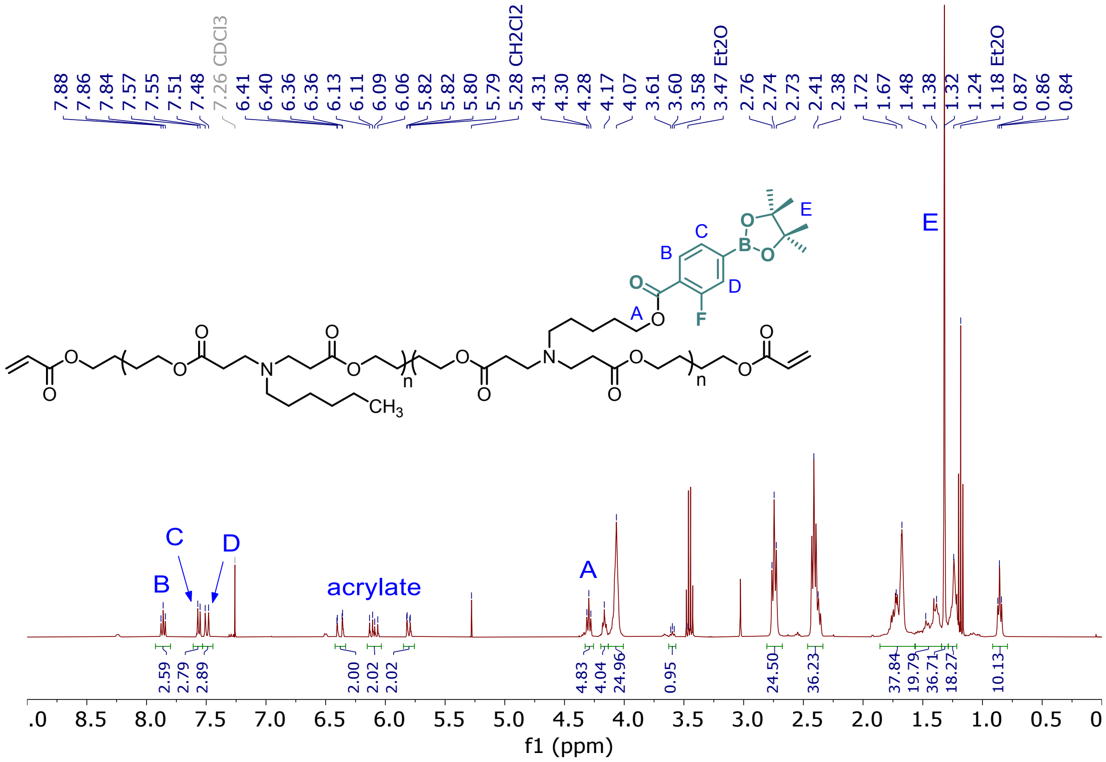

Figure S15. Spectroscopic characterization of **C6-FPBpin** poly(β-aminoester): ^{1}H-NMR in $CHCl_3$-d_1.

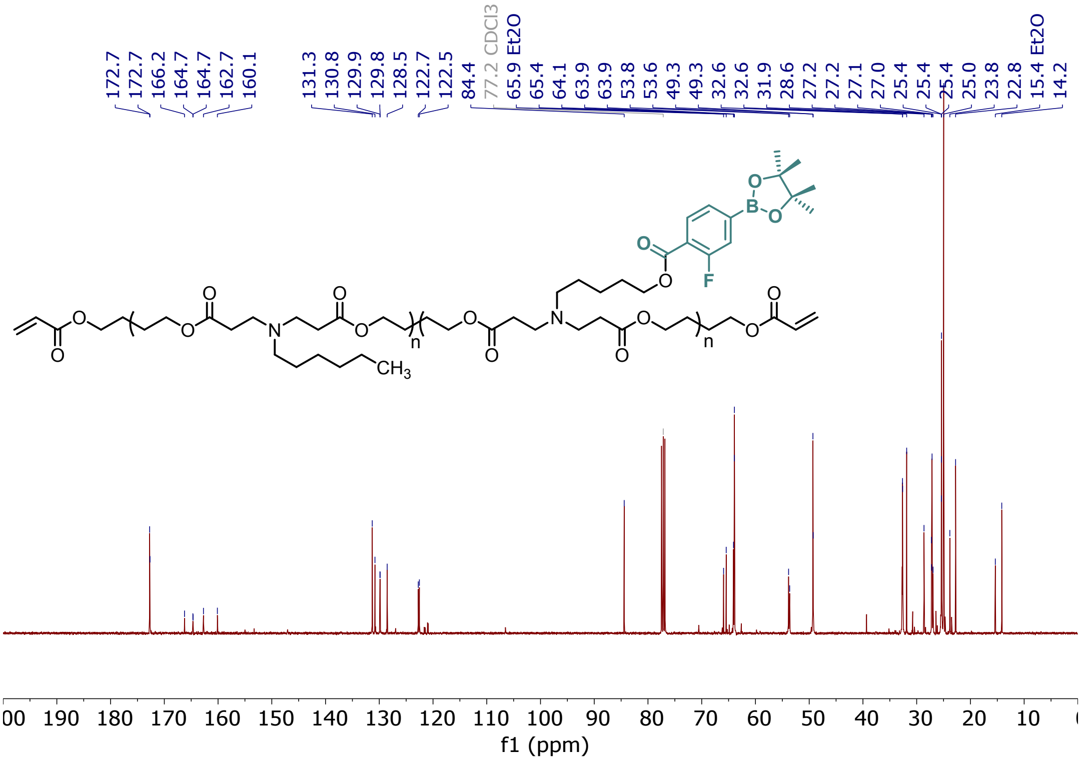

Figure S16. Spectroscopic characterization of **C6-FPBpin** poly(β-aminoester): ^{13}C-NMR in $CHCl_3$-*d_1*.

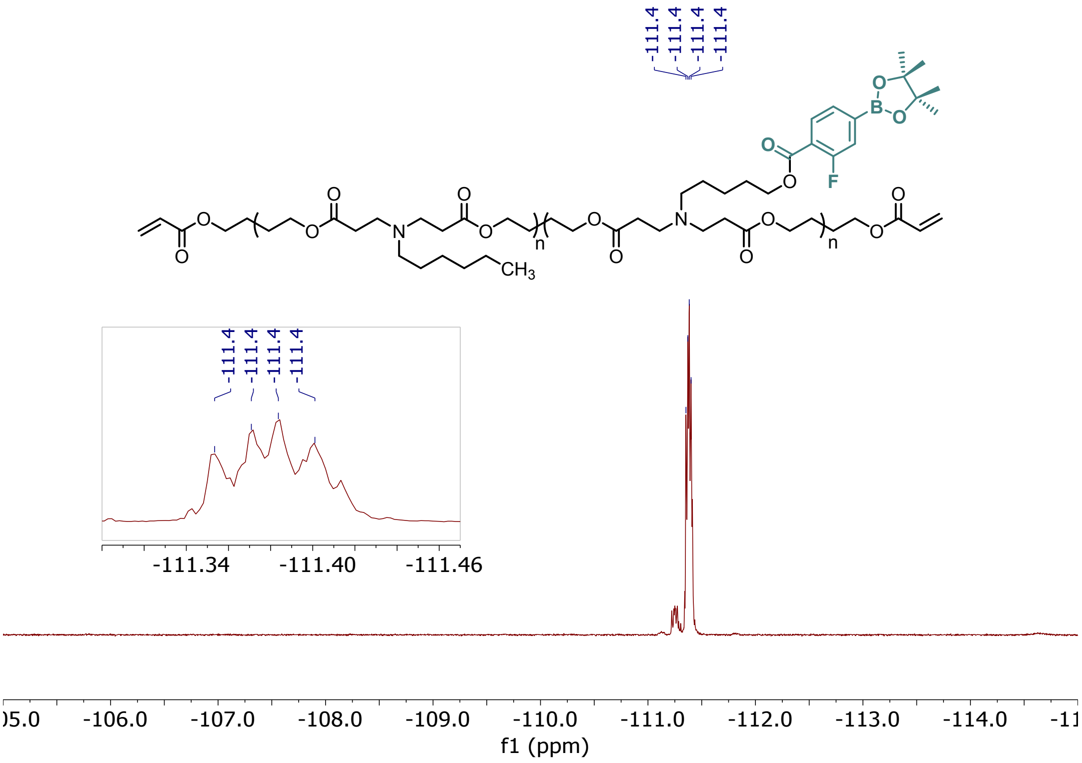

Figure S17. Spectroscopic characterization of **C6-FPBpin** poly(β-aminoester): ^{19}F-NMR in $CHCl_3$-*d_1*.

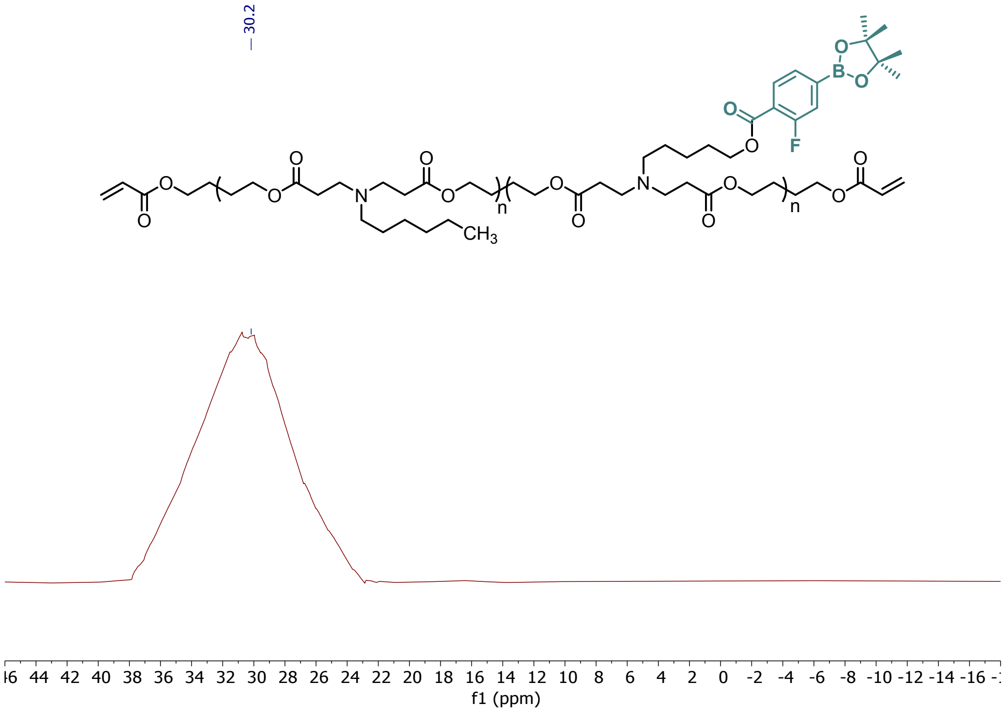

Figure S18. Spectroscopic characterization of **C6-FPBpin** poly(β-aminoester): ^{11}B-NMR in $CHCl_3$-d_1.

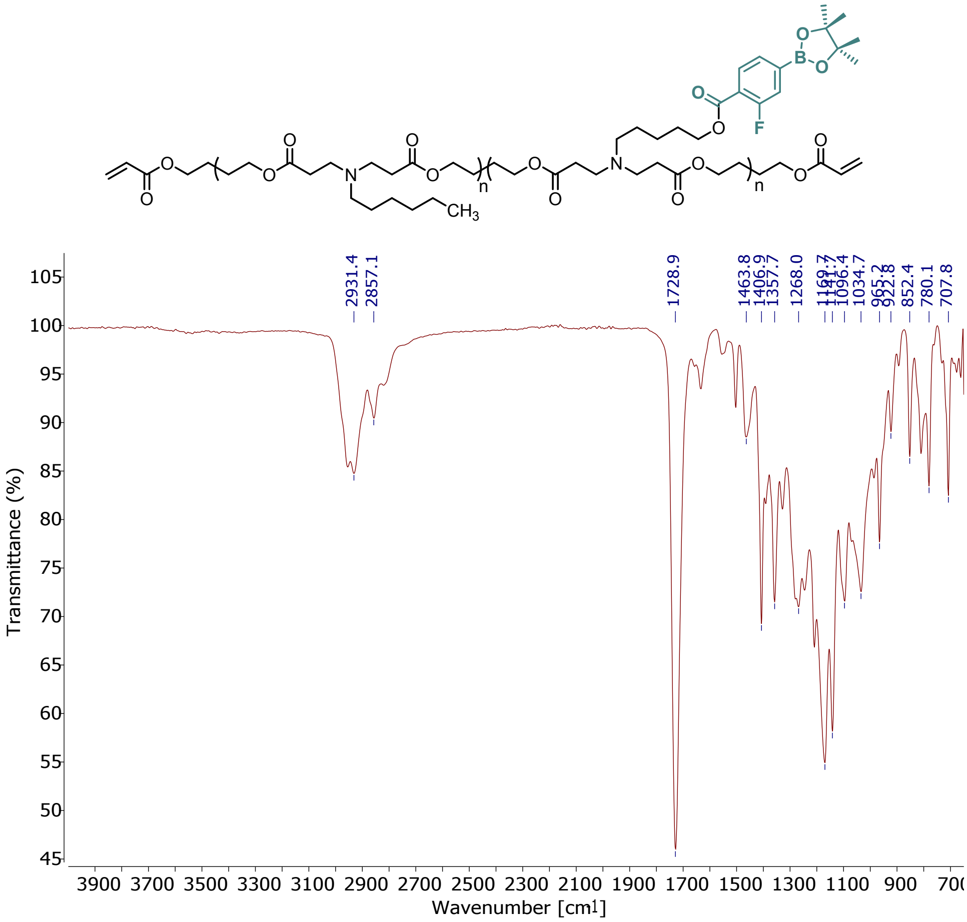

Figure S19. Spectroscopic characterization of **C6-FPBpin** poly(β-aminoester): FTIR by ATR (neat).

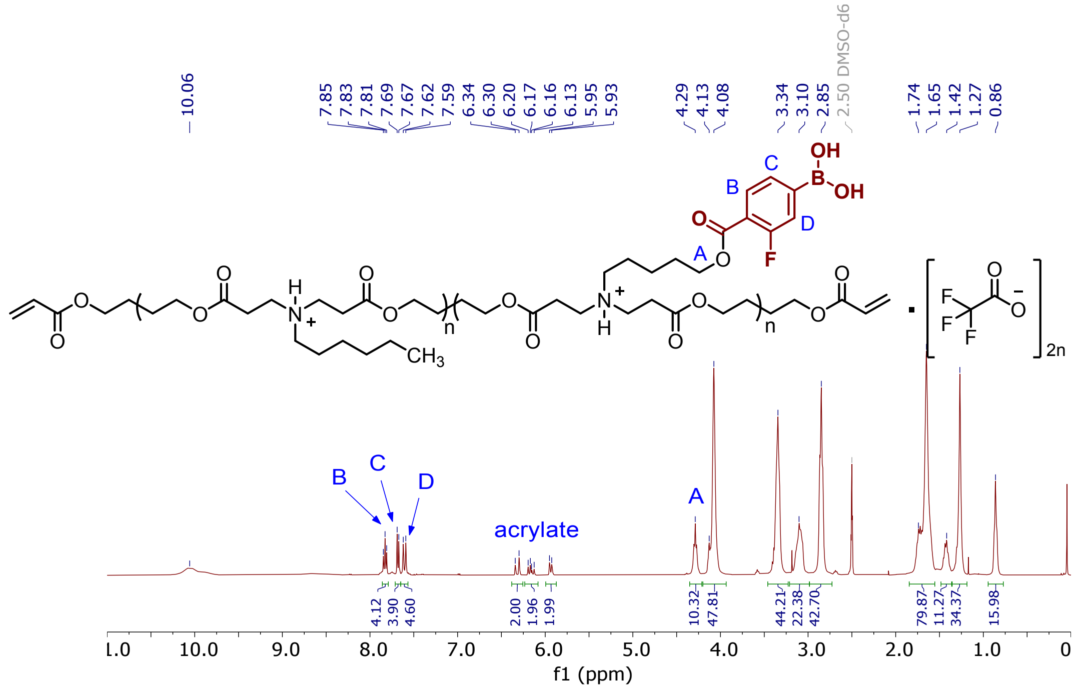

Figure S20. Spectroscopic characterization of **C6-FPBA** poly(β-aminoester): ^{11}B-NMR in DMSO-d_6.

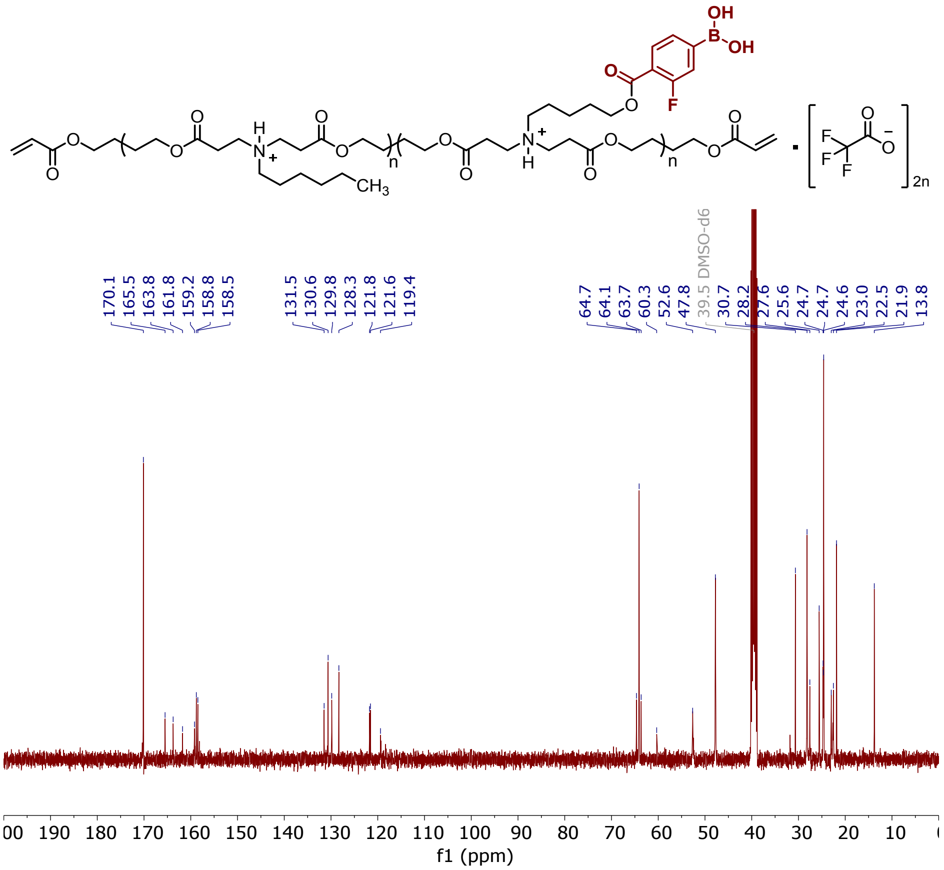

Figure S21. Spectroscopic characterization of **C6-FPBA** poly(β-aminoester): ^{13}C-NMR in DMSO-d_6.

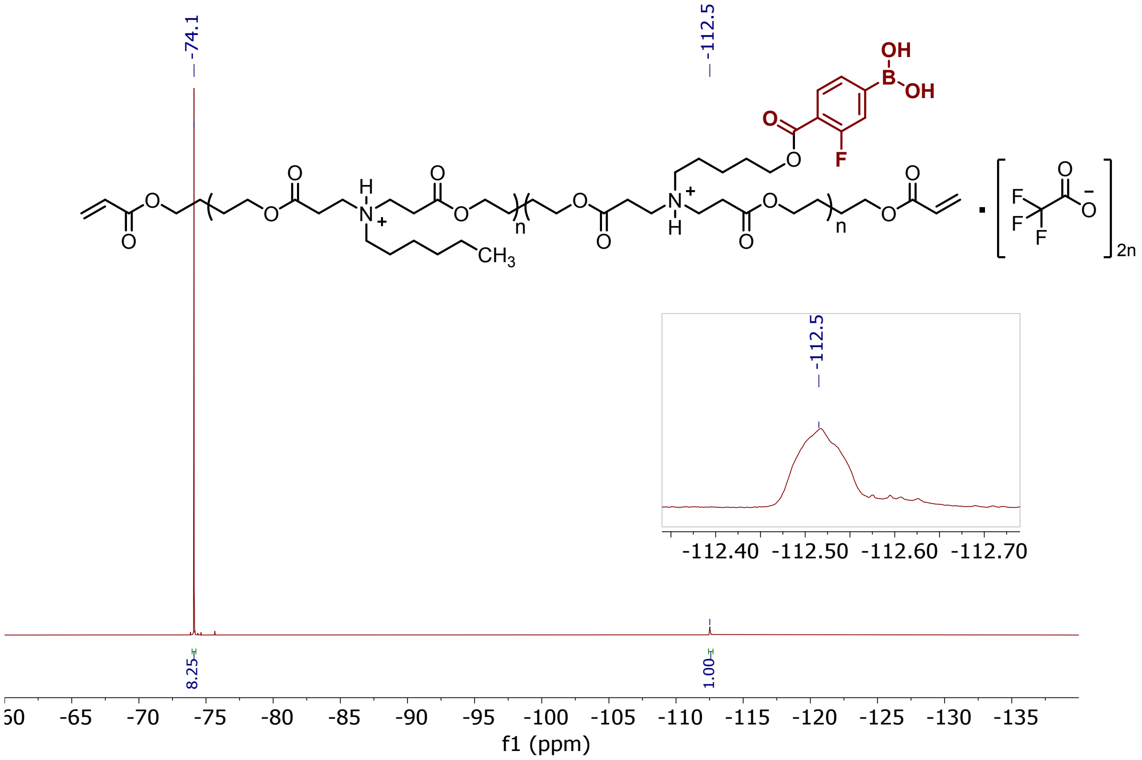

Figure S22. Spectroscopic characterization of **C6-FPBA** poly(β-aminoester): ^{19}F-NMR in DMSO-d_6.

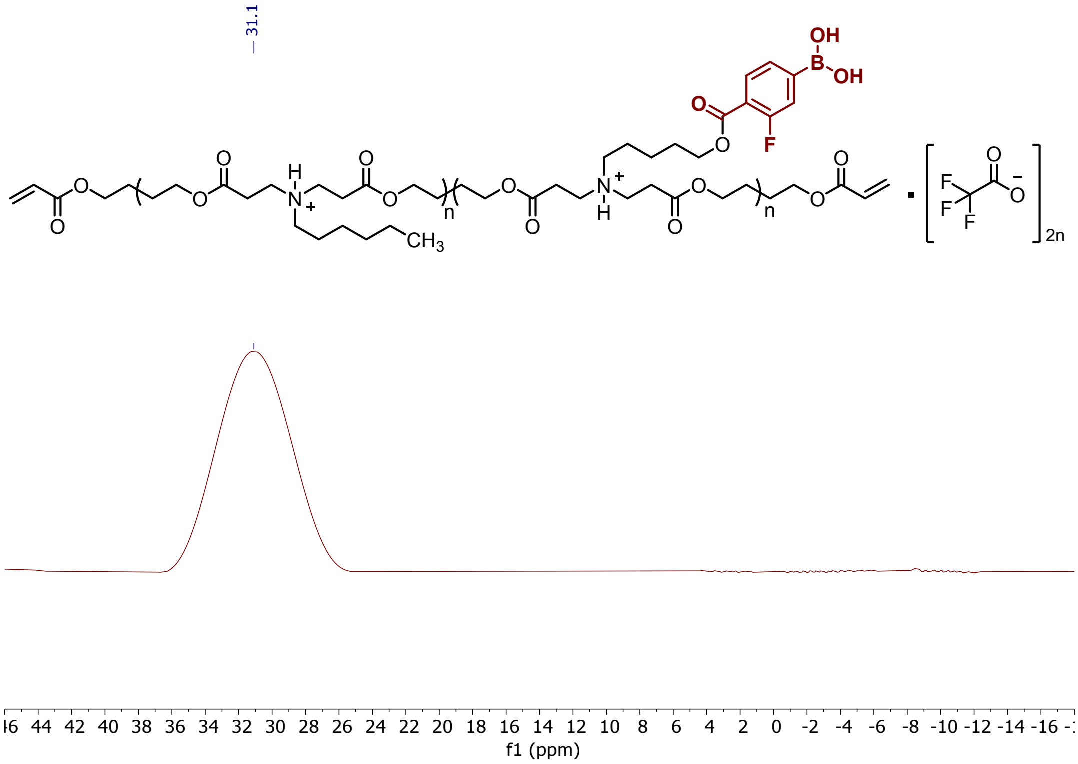

Figure S23. Spectroscopic characterization of **C6-FPBA** poly(β-aminoester): ^{11}B-NMR in DMSO-d_6.

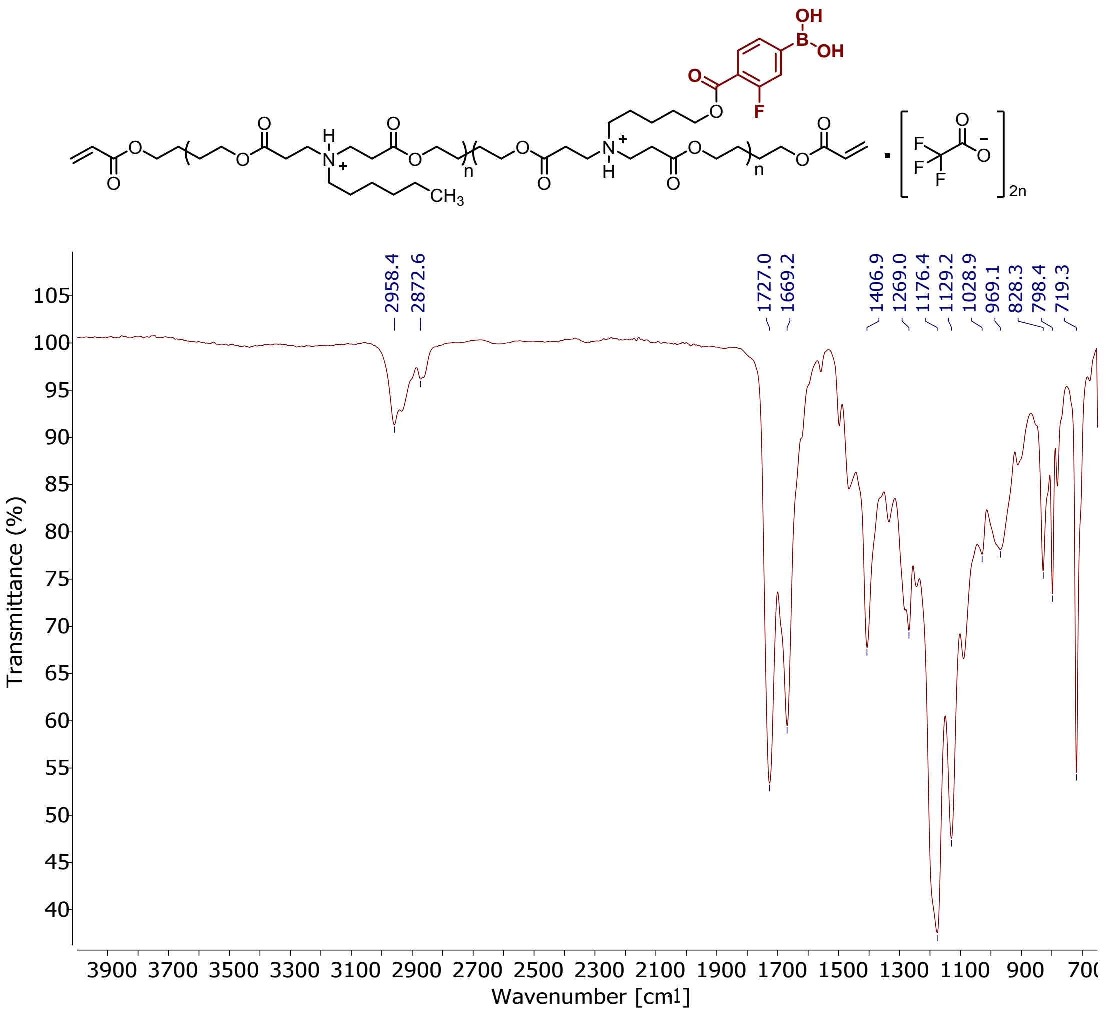

Figure S24. Spectroscopic characterization of **C6-FPBA** poly(β-aminoester): FTIR by ATR (neat).

(**C6**·TFA): ^{1}H NMR (DMSO-d_6, 400 MHz) δ 6.32 (dd, *J* = 17.3, 1.7 Hz, 2H), 6.16 (dd, *J* = 17.3, 10.3 Hz, 2H), 5.94 (dd, *J* = 10.2, 1.8 Hz, 2H), 4.39 (t, *J* = 6.4 Hz, 5H), 4.17 – 4.00 (m, 30H), 3.35 (t, *J* = 7.4 Hz, 25H), 3.17 – 3.03 (m, 12H), 2.92 – 2.79 (m, 25H), 1.80 – 1.56 (m, 48H), 1.50 – 1.21 (m, 28H), 0.90 – 0.82 (m, 10H).

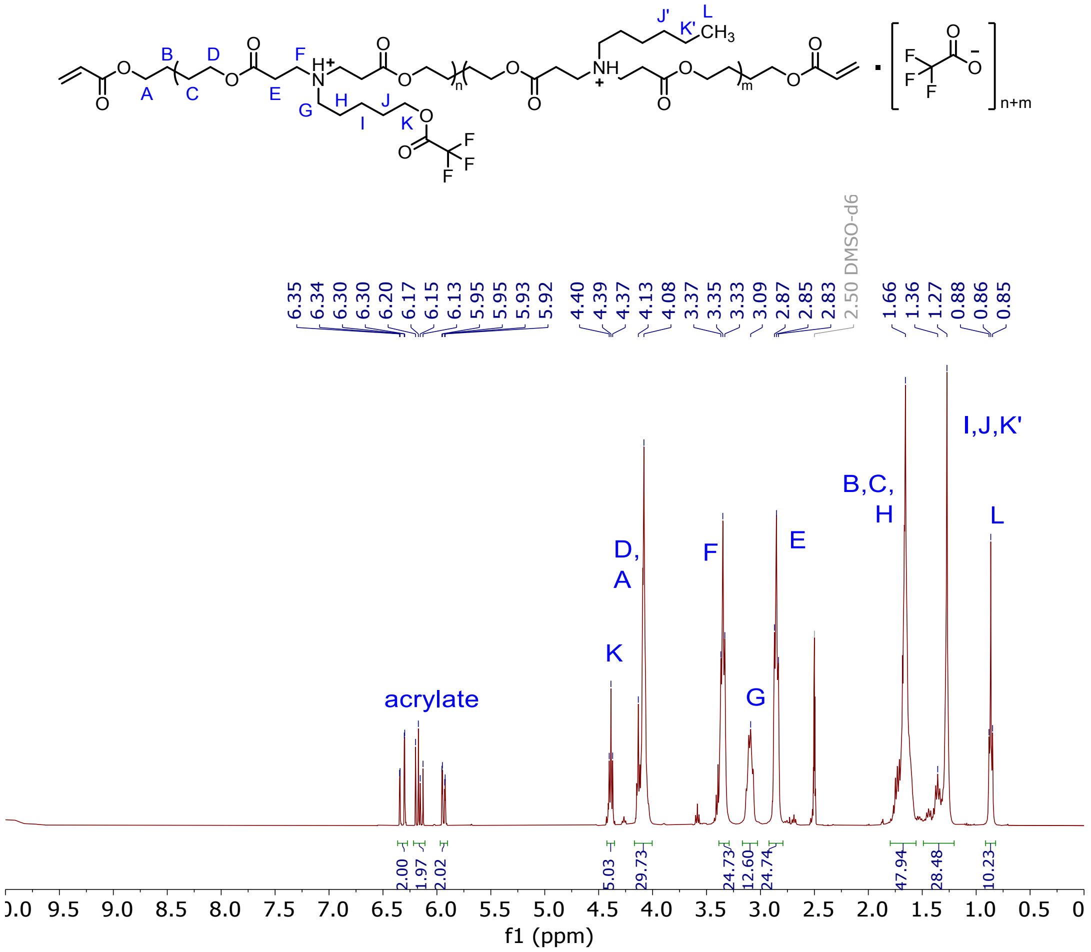

Figure S25. ^{1}H NMR control spectra (in DMSO-d_6) of the diacrylate-terminated poly(β-aminoester) **C6** upon treatment with trifluoroacetic acid (TFA). PBAE **C6**·TFA was prepared evaporating *in vacuo* a solution of PBAE **C6** at 100 mg/mL in a mixture of CH_2Cl_2 with 5 % TFA. Under these conditions, the hydroxyl groups of **C6** are esterified and the amine-backbone are found protonated with trifluoroacetate as counterion.

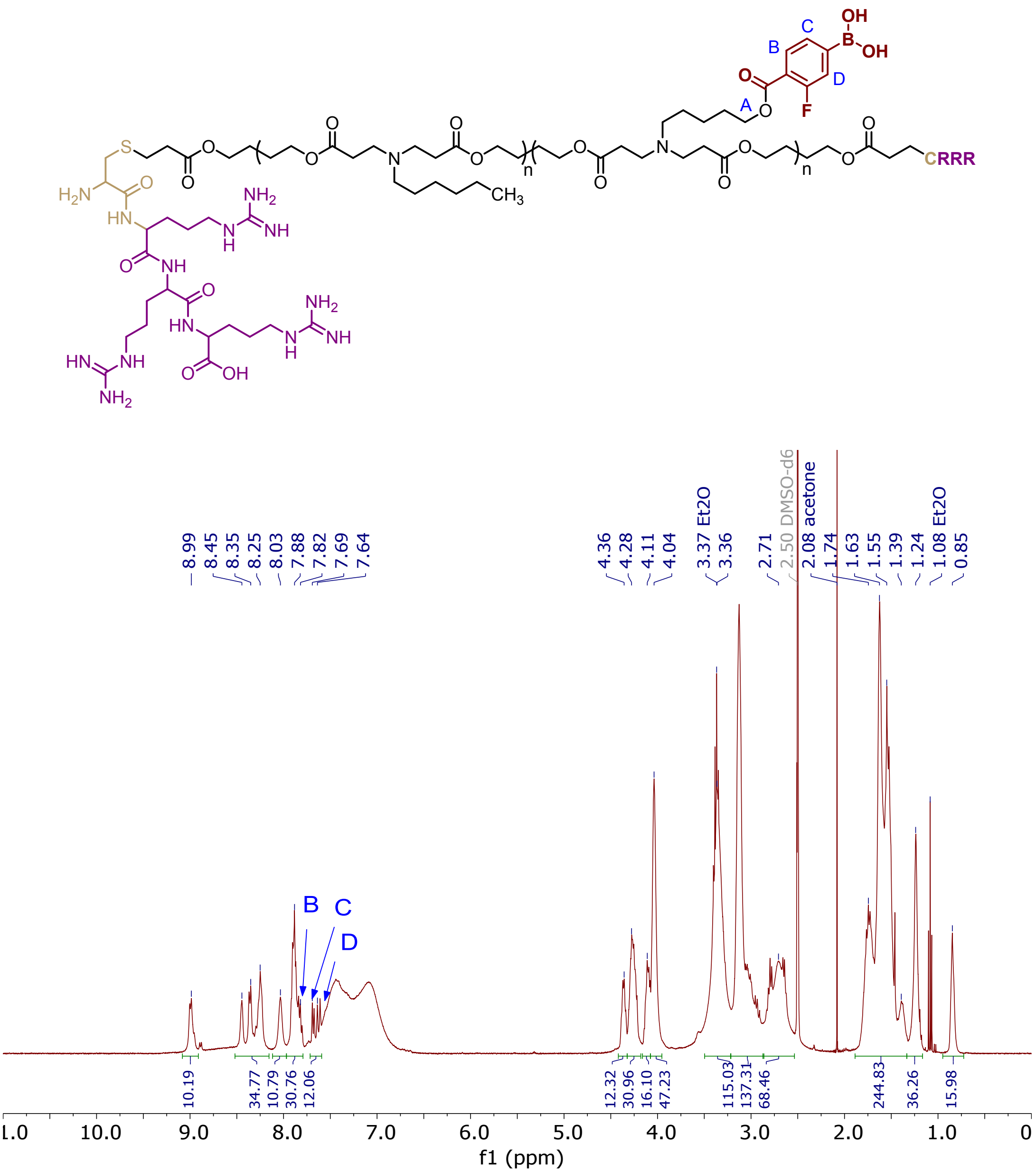

Figure S26. Spectroscopic characterization of **C6-FPBA-C$_2$R$_6$** (**Bor-pBAE**): ^{1}H-NMR in DMSO-d_6.

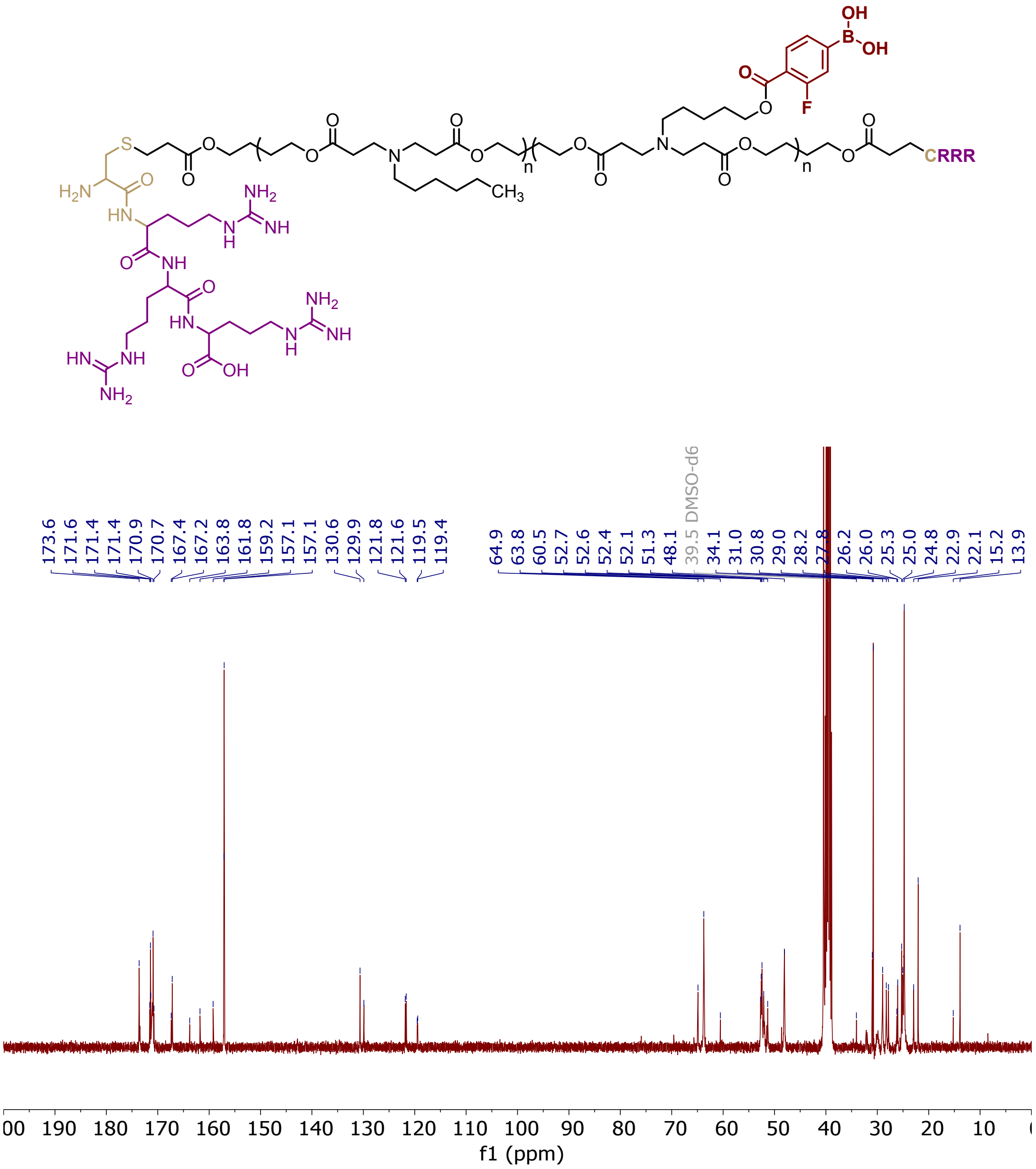

Figure S27. Spectroscopic characterization of **C6-FPBA-C_2R_6** (**Bor-pBAE**): ^{13}C-NMR in DMSO-d_6.

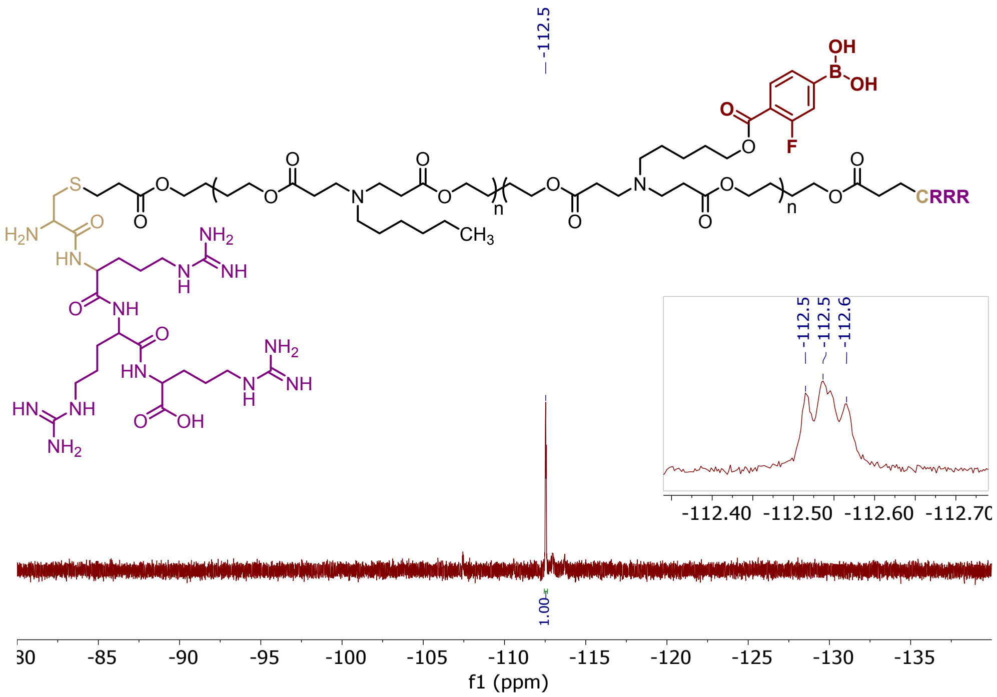

Figure S28. Spectroscopic characterization of **C6-FPBA-C$_2$R$_6$** (**Bor-pBAE**): ^{19}F-NMR in DMSO-d_6.

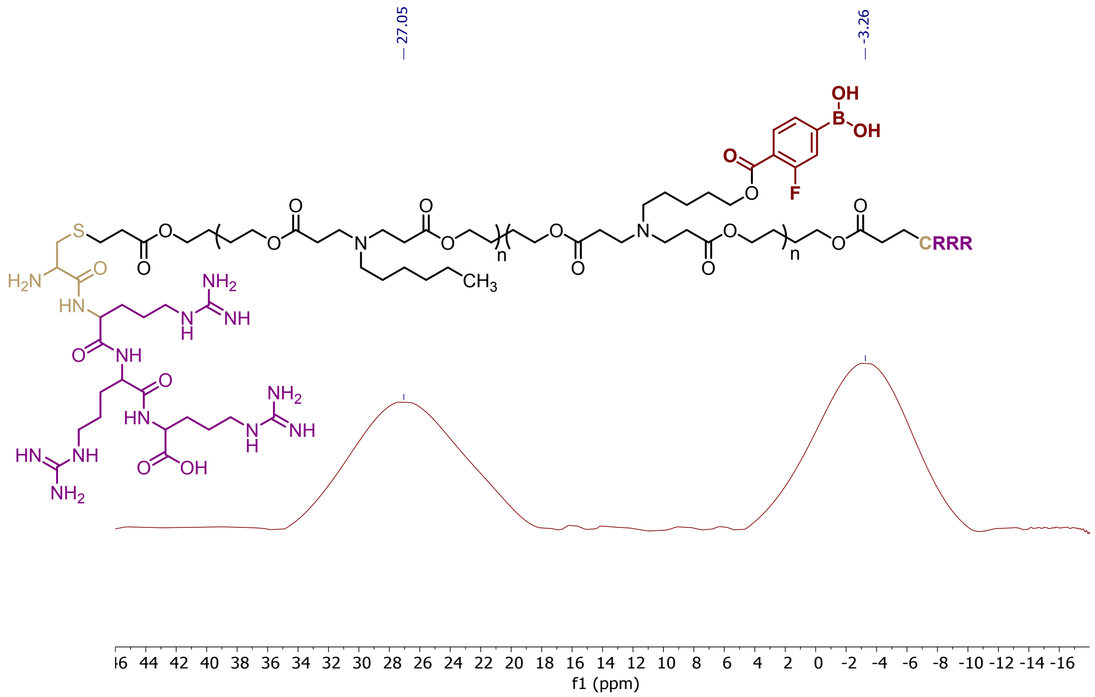

Figure S29. Spectroscopic characterization of **C6-FPBA-C$_2$R$_6$** (**Bor-pBAE**): ^{11}B-NMR in DMSO-d_6.

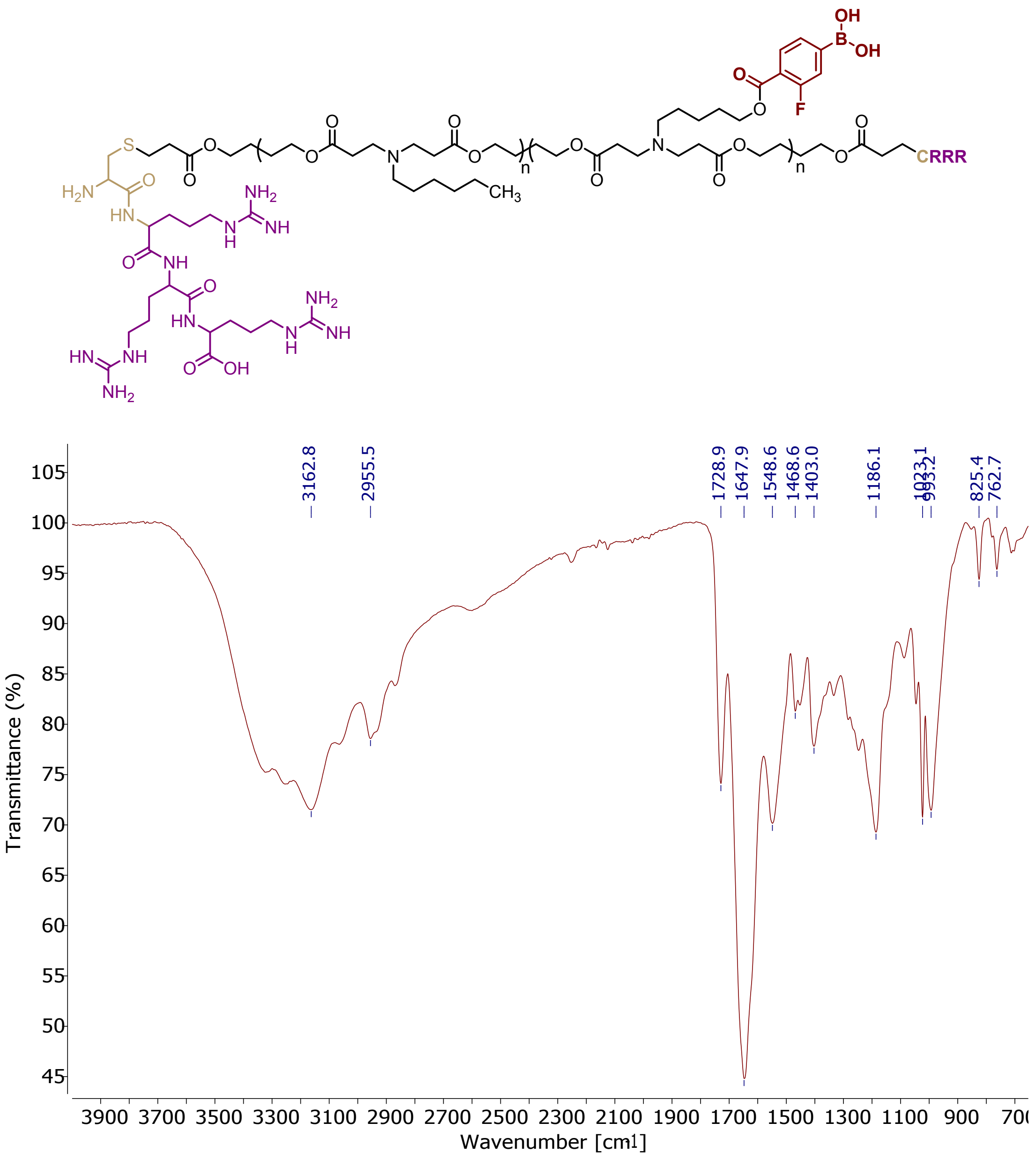

Figure S30. Spectroscopic characterization of **C6-FPBA-C$_2$R$_6$** (**Bor-pBAE**): FTIR by ATR (neat).

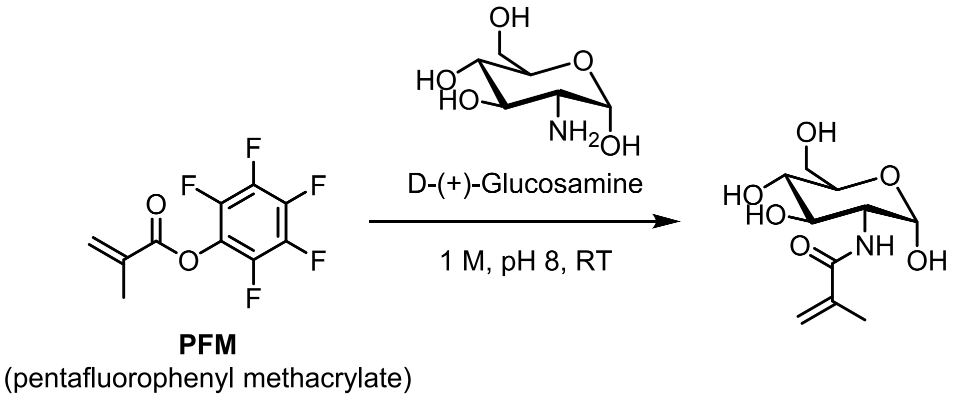

Figure S31. Scheme of the nucleophilic substitution reaction between the active ester of PFM and amine of glucosamine. RT: Room Temperature.

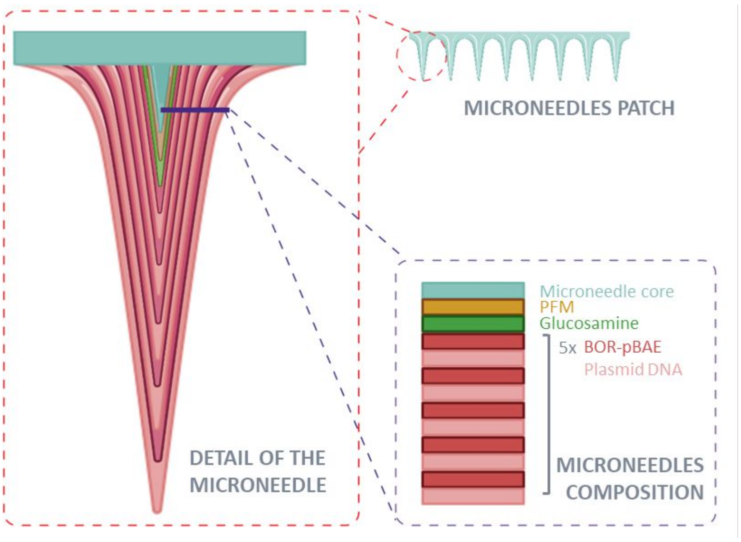

Figure S32. Scheme of the multilayered coating over the microneedles devices. The coating is composed by a first layer of PFM (PentaFluorophenylMethacrylate), a second layer of glucosamine and 10 layers of alternate components: Bor-pBAE polymer and pGFP (DNA Plasmid).

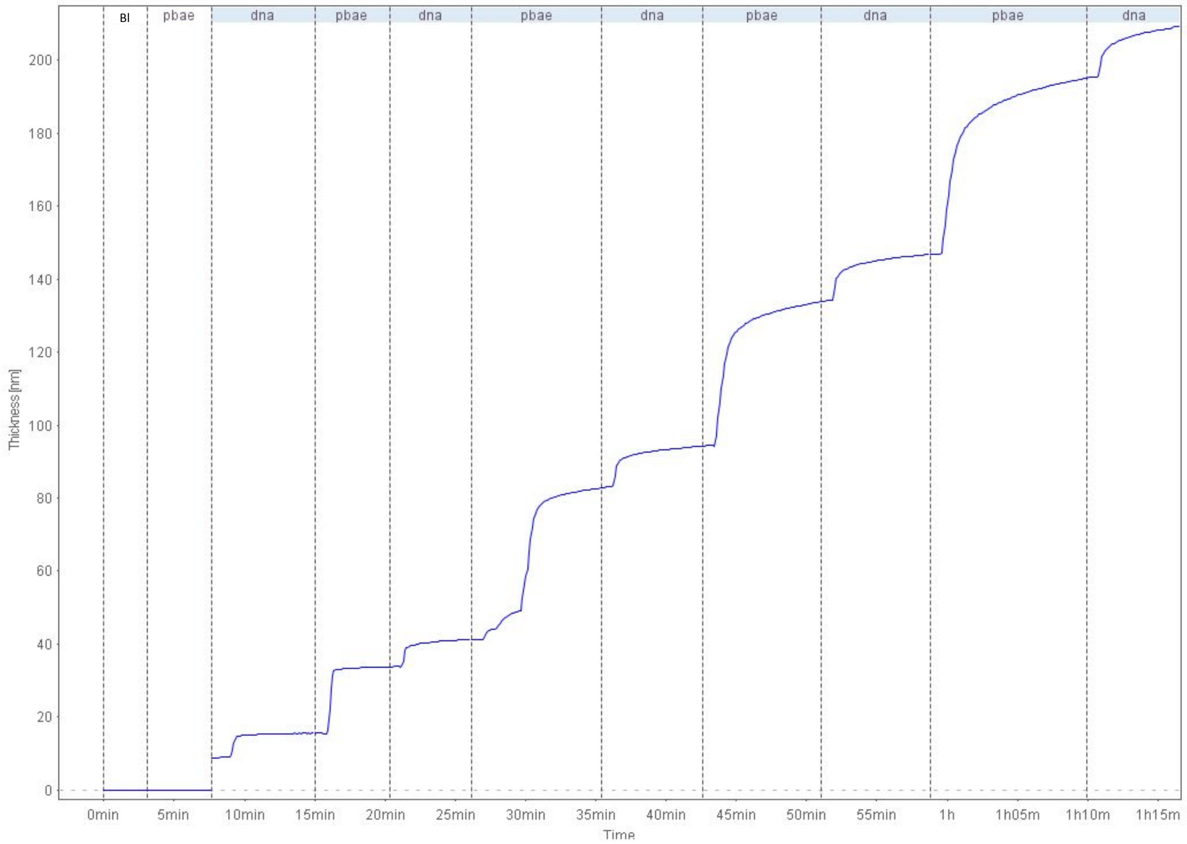

Figure S33. Thickness of the 10 layers PEM system coating pp-PFM/Glucosamine sensor (layers pH8), obtained through Sauerbrey model calculations. Bl=Baseline.

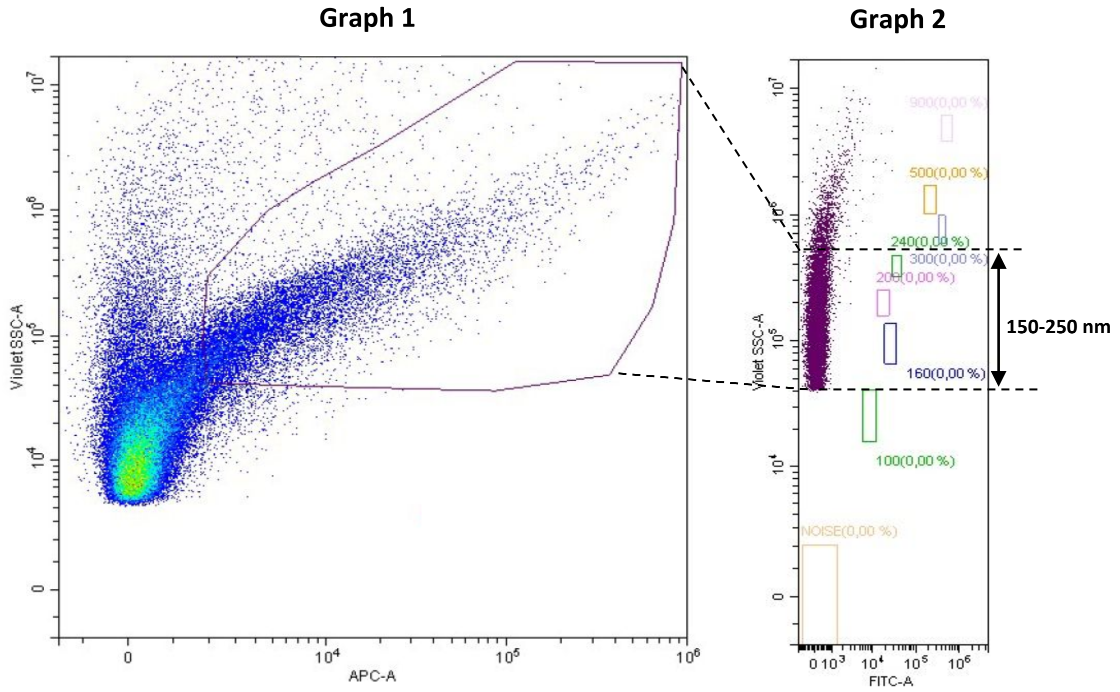

Figure S34. Establishment of the range of size of Cy5+ Bor-pBAE nanoparticles. This range was determined by comparing the gated region of Cy5 positive particles (graph 1 and purple dot plot of graph 2) with the analysis

of fluorescence beads of different diameters which act as reference (regions of different sizes located on the right side of the purple population in graph 2).

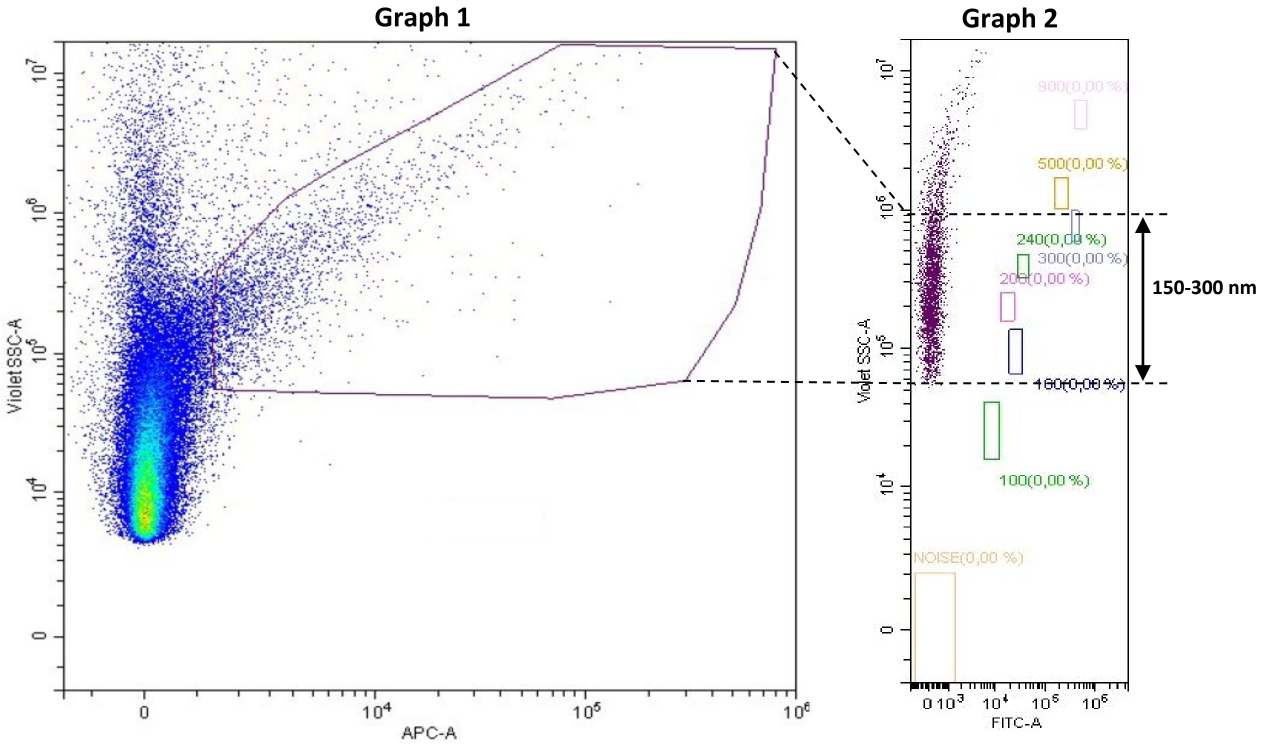

Figure S35. Establishment of the range of size of Cy5+ Bor-pBAE nanoparticles released at pH= 7.4. This range was determined by comparing the gated region of Cy5 positive particles (graph 1 and purple dot plot of graph 2) with the analysis of fluorescence beads of different diameters which act as reference (regions of different sizes located on the right side of the purple population in graph 2).

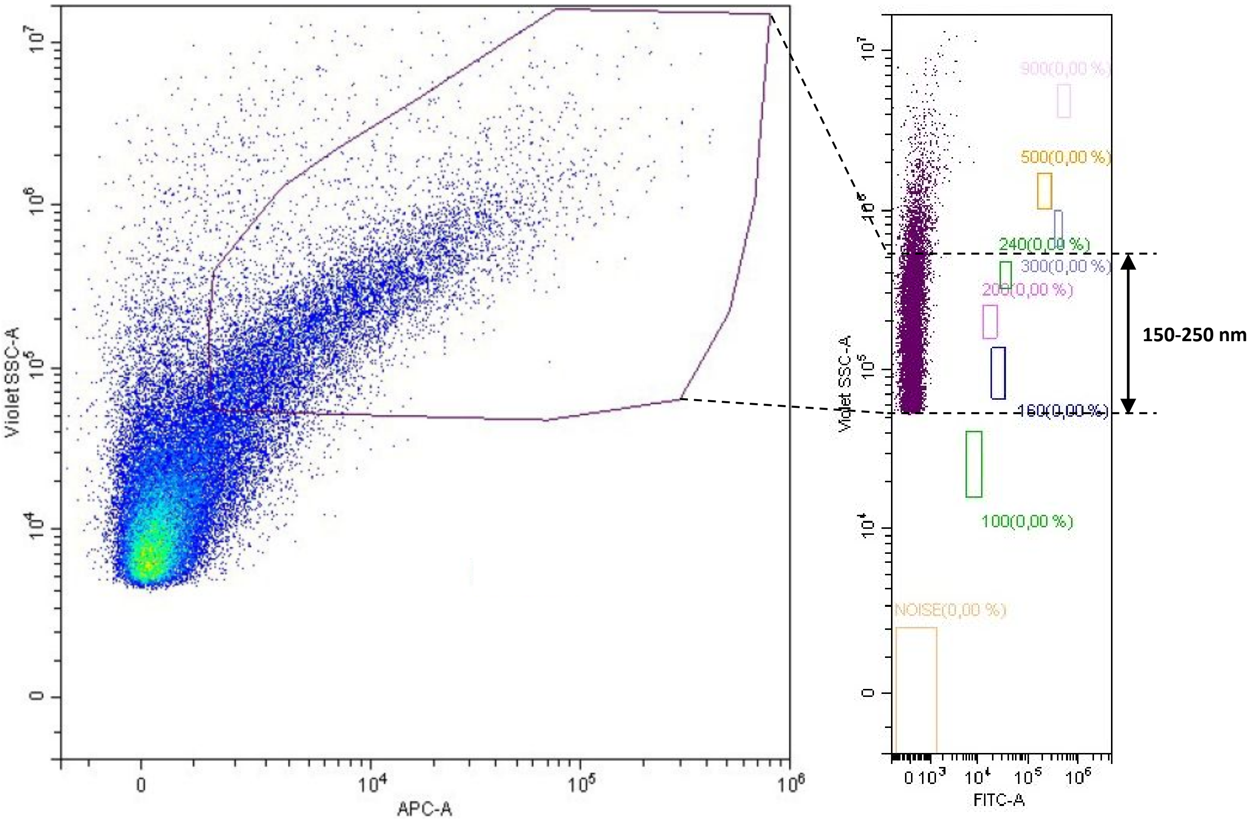

Figure S36. Establishment of the range of size of Cy5+ Bor-pBAE nanoparticles released at pH= 5.1. This range was determined by comparing the gated region of Cy5 positive particles (graph 1 and purple dot plot of graph 2) with the analysis of fluorescence beads of different diameters which act as reference (regions of different sizes located on the right side of the purple population in graph 2).

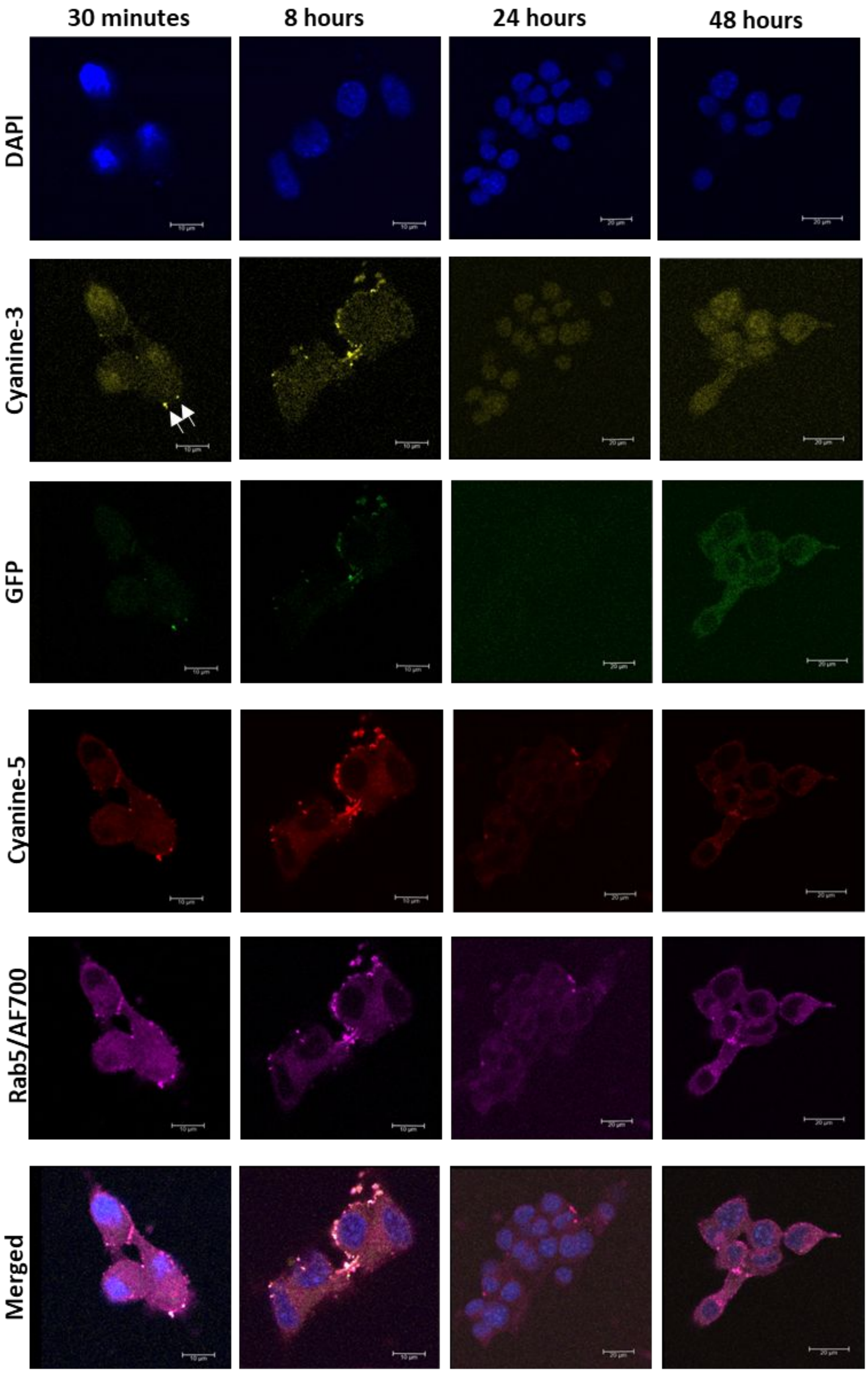

Figure S37. Complete panel for the confocal micrographs of the NPs uptake.

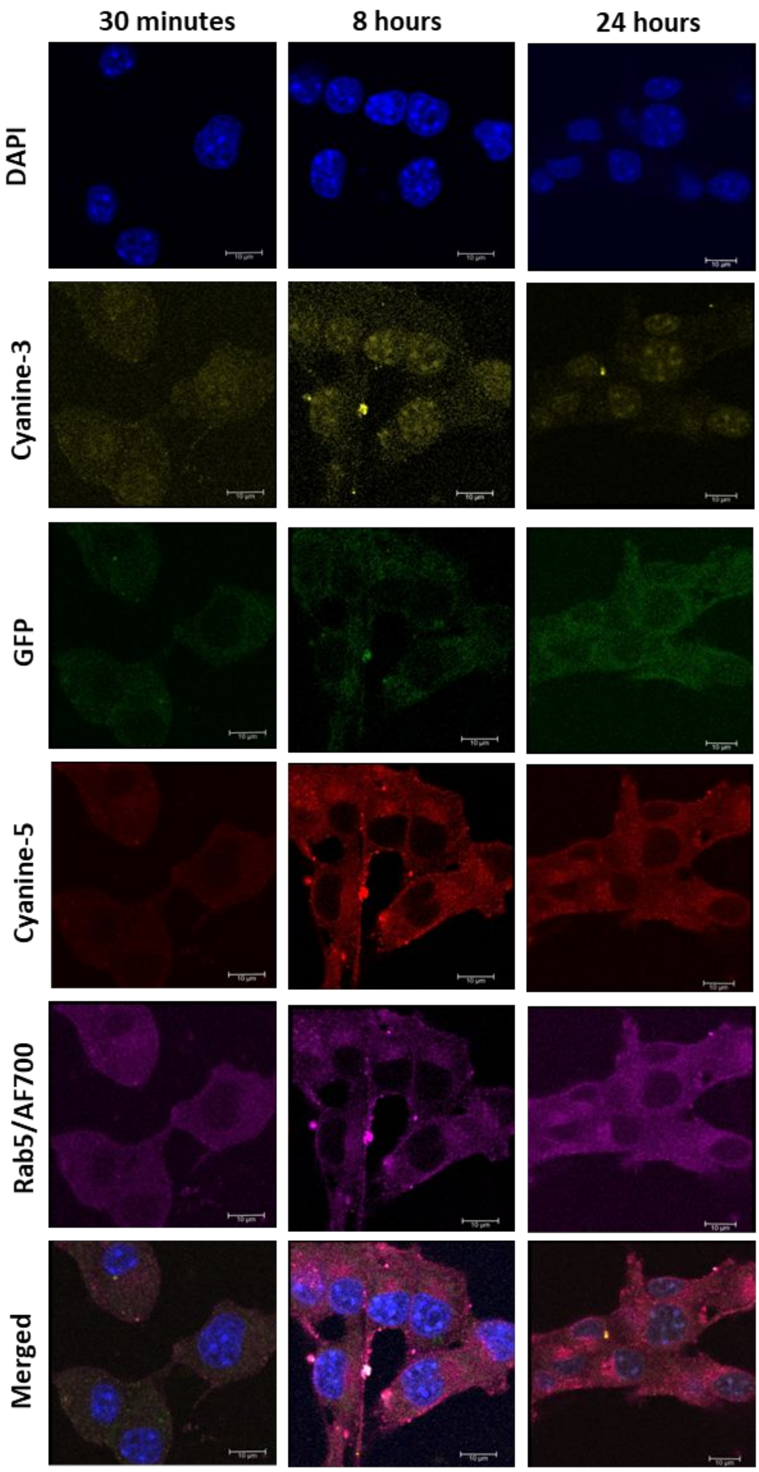

Figure S38. Complete panel of the confocal micrographs of the multilayers release at pH=5.1.

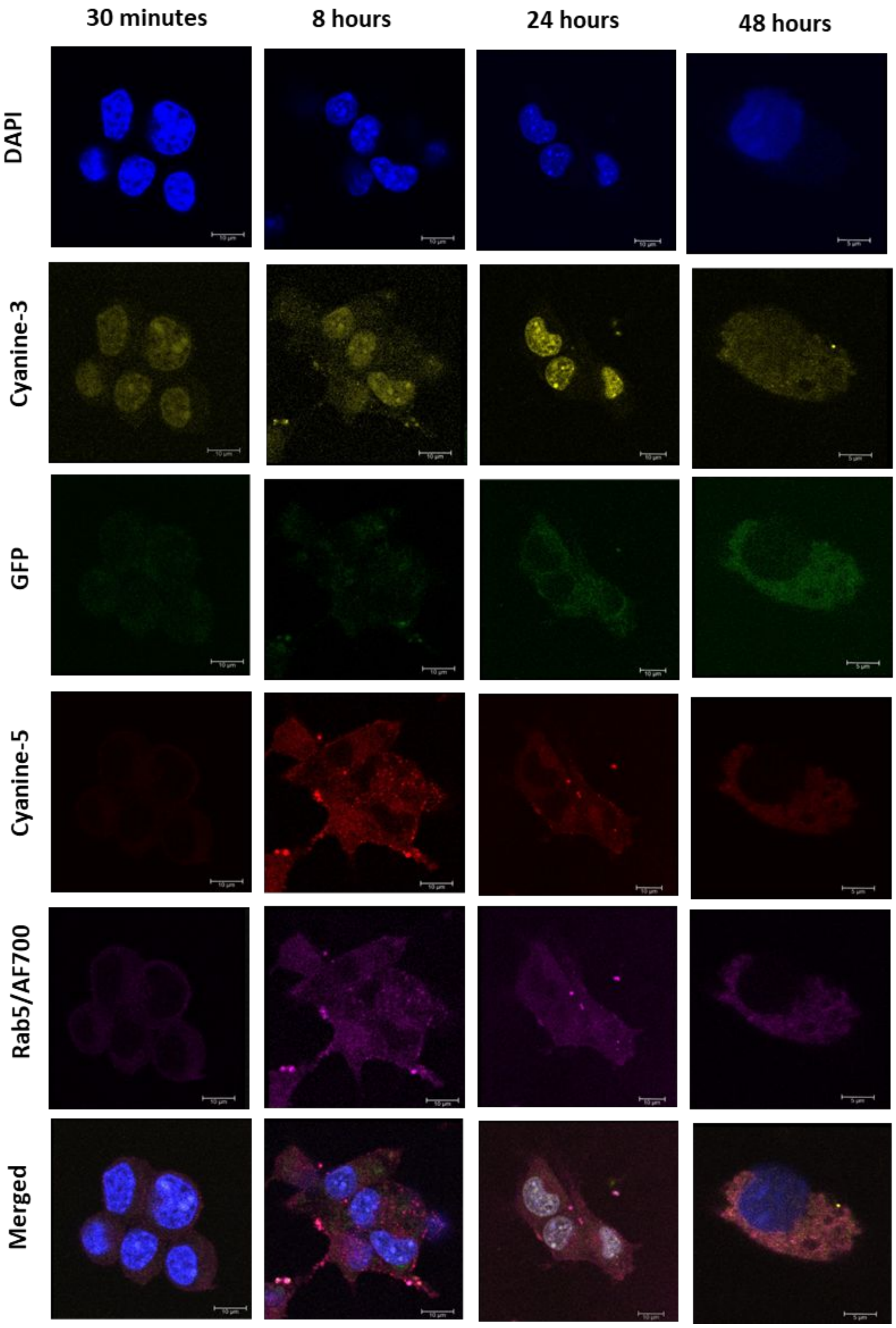

Figure S39. Complete panel of the confocal micrographs of the multilayers release at pH=7.4.
